# Small-molecules that covalently react with a human prolyl hydroxylase – towards activity modulation and substrate capture†
†Electronic supplementary information (ESI) available: Synthesis, mass spectrometry, inhibition assays, mutagenesis. See DOI: 10.1039/c8cc07706a


**DOI:** 10.1039/c8cc07706a

**Published:** 2018-11-19

**Authors:** Jacob T. Bush, Robert K. Leśniak, Tzu-Lan Yeh, Roman Belle, Holger Kramer, Anthony Tumber, Rasheduzzaman Chowdhury, Emily Flashman, Jasmin Mecinović, Christopher J. Schofield

**Affiliations:** a Chemistry Research Laboratory , University of Oxford , 12 Mansfield Road , Oxford , OX1 3TA , UK . Email: christopher.schofield@chem.ox.ac.uk; b Institute for Molecules and Materials , Radboud University , Heyendaalseweg 135 , 6525 AJ Nijmegen , The Netherlands; c Department of Physics, Chemistry and Pharmacy , University of Southern Denmark , Campusvej 55 , 5230 Odense , Denmark . Email: mecinovic@sdu.dk

## Abstract

We describe covalently binding modulators of the activity of human prolyl hydroxylase domain 2 (PHD2) and studies towards a strategy for photocapture of PHD2 substrates. Reversible active site binding of electrophile bearing compounds enables susbsequent covalent reaction with a lysine residue (K408) in the flexible C-terminal region of PHD2 to give a modified protein that retains catalytic activity.

## Abstract 2

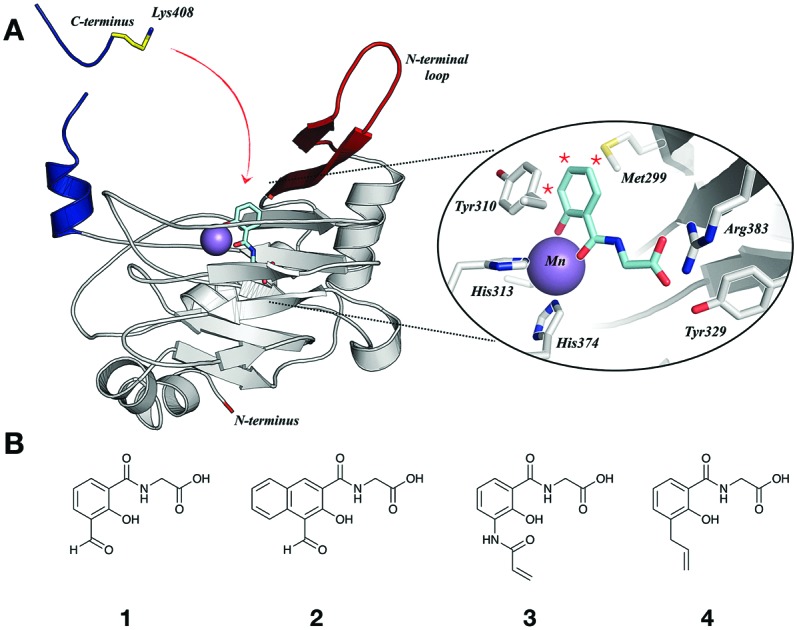
We describe covalently binding modulators of the activity of human prolyl hydroxylase domain 2 (PHD2) and studies towards a strategy for photocapture of PHD2 substrates.

## 

The hypoxia inducible factor (HIF) prolyl hydroxylases (PHD1-3) play a central role in cellular responses to hypoxia in humans and other animals.^1^,^2^ PHD-catalysed prolyl hydroxylation of HIF-α isoforms (Pro402, Pro564 in human HIF-1α) signals for proteasomal degradation, hence hindering formation of transcriptionally active α,β-HIF. When oxygen levels are limiting, PHD catalysis slows and HIF-α levels rise, so enabling α,β-HIF mediated upregulation of a gene array working to alleviate hypoxic effects.^3^ The PHDs are Fe(ii) and 2-oxoglutarate (2OG) dependent oxygenases. PHD inhibitors are in clinical trials for anaemia treatment because erythropoietin (EPO) is a HIF target gene. Clinical candidate PHD inhibitors, like most reported 2OG oxygenase inhibitors, are 2OG competitors coordinating to the active site Fe(ii).^4^–^7^ Since the identification of HIF-α as a PHD substrate, there have been reports of non-HIF PHD substrates, though the physiological roles of these are uncertain.^8^

Inspired by the mode of action of aspirin in modulating, but not ablating, cyclooxygenase activity by covalent modification,^9^ we are interested in developing molecules that change PHD activity rather than blocking catalysis. Such compounds have potential to alter PHD substrate selectivity and oxygen dependence. The former is medicinally relevant because HIF-1α and HIF-2α have different gene targets; *e.g.* anaerobic glycolysis is a HIF-1α target, whereas vascular endothelial growth factor (VEGF) is a HIF-2α target. We envisaged covalent modification of a non-catalytically essential PHD component may alter the oxygen dependence of the PHDs, ultimately enabling safer medicines than those working by complete inhibition. Covalent inhibition may provide a strategy for selective inhibition by reacting with non-conserved residues. The ability to install a reactive functional group near the active site, without inhibition, may also enable substrate capture. Here we communicate initial findings demonstrating the feasibility of selective covalent modification of human PHD2.

Covalent conjugation of small molecules to proteins is often achieved by reaction of an electrophile with a nucleophilic residue.^10^–^13^ PHD2 structures suggest that its active site does not contain Lys- or Cys-residues; however, Lys-residues are present in a loop (K246, K251) and the C-terminal section (K400, K402, K408, K416, K423), both of which are flexible and associated with the active site entrance (Fig. 1A).^14^,^15^ We envisioned appropriate functionalisation of a PHD2 inhibitor with an electrophile may enable covalent reaction with one of these Lys-residues. We proposed that the flexibility of these regions might enable the covalently bound inhibitor to associate and dissociate from the active site, causing activity modulation rather than complete inhibition.

**Fig. 1:**
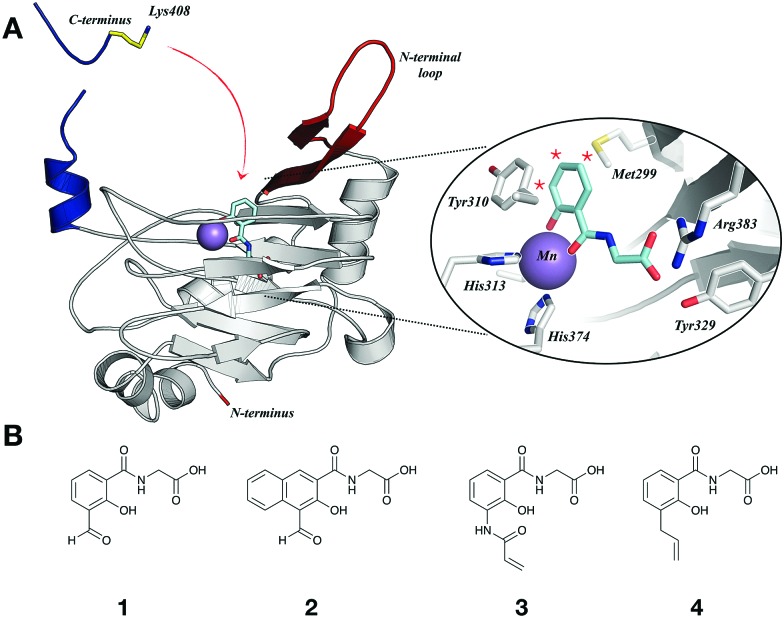
(A) View from a crystal structure of PHD2 in complex with Mn (purple, substituting for Fe) docked with a bidentate inhibitor, highlighting the flexible C-terminal tail (blue) and loop (red). Inset: Close-up active site view. (B) Salicylic acid derivatives as potential covalent inhibitors of PHD2, functionalised either with an aldehyde (**1** and **2**) or a Michael acceptor (**3**), and a control, non-covalently binding inhibitor (**4**).

Salicylic and picolinic acid derivatives coupled to glycine are reversible PHD2 inhibitors.^5^ Modelling based on structures, including of the catalytic domain of PHD2 with *N*-oxalylglycine (NOG) (Fig. 1A), indicated that an electrophile at the 3, 4 or 5 positions of the salicylic acid aryl ring may be positioned to react with the targeted flexible regions. Initially, two aldehyde derivatives of glycine-coupled salicylic acid, **1** and **2**, along with several other analogues, were synthesised (Fig. 1B and Fig. S1, ESI†).

The interaction of the ligands with PHD2_181–426_ (hereafter PHD2) was examined by non-denaturing ESI-MS, using cone voltage variation to distinguish covalent and non-covalent complexes (non-covalent interactions are typically preserved at cone voltages of 50–80 V, and dissociated at 200 V).^16^–^18^ Aldehydes **1** and **2** were incubated with equimolar PHD2 and Fe(ii) (37 °C; 5 or 20 min). After 20 min, analysis at 200 V cone voltage indicated **1**/**2** react with PHD2 to give a peak with mass of PHD2 + **1**/**2** – H_2_O, suggesting condensation (Fig. 2). Analysis of **1** after 5 min indicated complete condensation; with **2**, reaction was slower, with an apparent non-covalent adduct predominating after 5 min, with ∼30% condensation (Fig. S2, ESI†). No peaks were observed for addition of more than one molecule of **1**/**2** to PHD2, implying site selective crosslinking (Fig. 2B). Without Fe(ii), neither covalent nor non-covalent adducts were observed, implying covalent conjugation involves active site Fe(ii) binding, with subsequent condensation. Consistent with this, preincubation with the reversible iron-binding inhibitor **FG2216** (IC_50_ < 1 μM),^15^ followed by addition of **1**/**2** did not manifest covalent adducts. No covalent modification was observed with the 5-formyl derivative (S1) or an analogue lacking the glycine sidechain (S2) (Fig. S3 and S4, ESI†). Some apparent covalent modification was observed with a ketone analogue of **1** (S3) and with a pyridinyl analogue (S4), but to a lesser extent than for **1**/**2** (Fig. S4, ESI†).

**Fig. 2:**
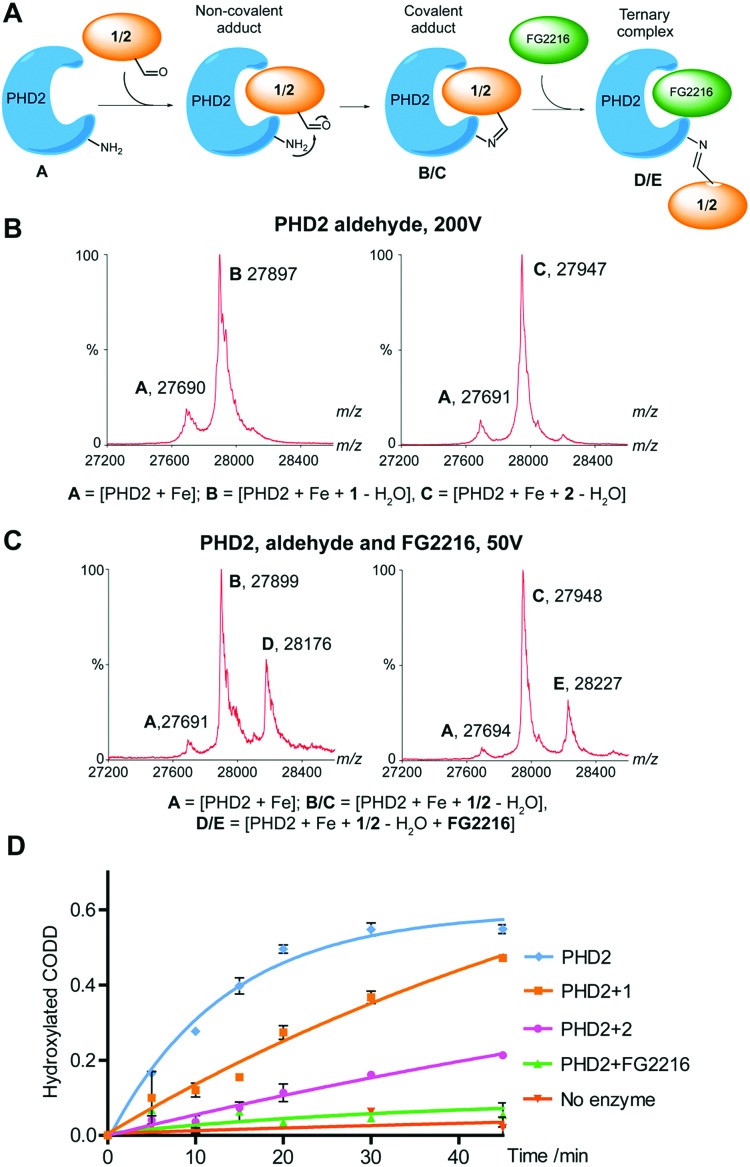
(A) Scheme showing species formed on PHD2 incubation with **1**/**2**, and **FG2216**. (B) Non-denaturing MS spectra indicating **1**/**2** covalently crosslink with PHD2 (20 μM, 37 °C, 5 or 20 min, cone voltage 200 V) with equimolar Fe(ii) and **1**/**2**. (C) **FG2216** binds to PHD2 covalently modified by **1**/**2**. PHD2 was incubated (37 °C, 20 min) with equimolar Fe(ii) and **1**/**2** to enable covalent reaction. Subsequently, **FG2216** (20 μM) was added, followed by a further incubation (37 °C, 5 min, cone voltage 50 V). (D) Evidence of CODD hydroxylation by covalently modified PHD2.

When **FG2216**^15^ (20 μM) was added to the pre-formed covalent adducts PHD2·Fe·**1** and PHD2·Fe·**2**, MS analysis indicated formation of a tertiary complex PHD2·**1**/**2**·**FG2216** (with Fe(ii)), suggesting the covalently-linked inhibitors can dissociate from the active site so enabling **FG2216** binding, while maintaining the covalent crosslink (Fig. 2C and Fig. S5, ESI†). The observation of a tertiary complex suggested that covalently crosslinked PHD2 may bind its substrate(s) and retain catalytic activity. The activity of covalently modified PHD2 was examined using MALDI-MS to monitor hydroxylation of the HIF-1α C-terminal oxygen dependent degradation domain (CODD, residues 556–574). Covalently modified PHD2 was prepared with complete (>95%) crosslinking as confirmed by MS and added to a solution of 2OG, ascorbate, CODD peptide and **1**/**2** (to maintain concentration of the inhibitor on dilution and so limiting hydrolysis). Controls were carried out without **1**/**2** and with **FG2216** (30 μM) (Fig. 2D). CODD hydroxylation was fastest without any inhibitors, and the activity was completely inhibited by **FG2216**. Interestingly, both PHD2·Fe·**1** and PHD2·Fe·**2** were apparently active, producing hydroxylated CODD slightly more slowly than unmodified PHD2. PHD2·Fe·**1** was more active than PHD2·Fe·**2**, consistent with the observation that **1** is more easily displaced by **FG2216** (Fig. S5, ESI†).

We next sought to create more stable covalent adducts by incorporation of an alternative electrophile. An analogous inhibitor with an α,β-unsaturated amide at C-3 of the salicylic acid ring was synthesised (**3**); we envisaged this may irreversibly react with a lysine residue by Michael addition (and so rule out the possibility of active PHD2 being produced by reversible covalent reaction). Compound **4**, without the amide, was synthesised as a negative control (Fig. S6, ESI†). **3**/**4** were incubated with equimolar PHD2/Fe(ii) and analysed by non-denaturing MS. After 5 min, spectra obtained at 50 V indicated the presence of PHD2·Fe·**3** and PHD2·Fe·**4**, which were assigned as non-covalent adducts by analysis at 200 V (Fig. S6, ESI†). Incubation for 1 and 2 h manifested ∼1/3 and 2/3 conversion, respectively, to the PHD2·Fe·**3** covalent adduct. No covalent adduct was observed with **4** after up to 2 h, supporting the proposed crosslinking to **3** by addition to the α,β-unsaturated amide (Fig. S6, ESI†). The slower rate of formation of the covalent adduct with **3** compared with aldehydes **1**/**2** may be due to the normally lower reactivity of α,β-unsaturated amides compared to aldehydes with amines.

In contrast to the likely imine adducts formed with **1**/**2**, the PHD2·Fe·**3** covalent adduct was stable to the acidic conditions of denaturing MS. This method was used for a timecourse study (Fig. S7, ESI†); near complete conversion to crosslinked PHD2·Fe·**3** occurred after 3 h (37 °C). The crosslinking could be fitted by a one-site decay function (*R*^2^ = 0.99, GraphPad Prism), consistent with a quasi intramolecular reaction of the PHD2·Fe·**3** non-covalent species to give a covalent adduct (Fig. S5, ESI†). The reaction rate was slower at lower temperatures, with little conversion at 4 °C (Fig. S8, ESI†).

To investigate whether the covalent PHD2·Fe·**3** adduct is active, PHD2 and **3** were pre-incubated (0, 1, 2 and 4 h) prior to assays. The extent of crosslinking was determined to be 0%, 50%, 66% and 90%, respectively (Fig. S9, ESI†). In controls lacking **3** during pre-incubation, resultant PHD2 activity decreased to the extent that fully inactive protein was manifested after 4 h pre-incubation. By contrast, the activity of PHD2 that was pre-incubated with **3**, remained as active as the non pre-incubated, uninhibited PHD2 for all pre-incubation time points, despite the fact that after 4 h pre-incubation with **3** the PHD2 was ∼90% covalently modified. This observation suggests that the covalently-linked ligand in PHD2·Fe·**3** reversibly (or does not) associates the active site, and can leave to allow the CODD substrate to bind.

PHD2 inhibition by **1**, **2** and **3** was measured using an antibody based assay to obtain IC_50_ values (Table S1, ESI†); all were weak inhibitors (IC_50_ > 100 μM), with **3** not reaching 50% inhibition at up to 2 mM. Interestingly, the IC_50_ values are >10 fold higher than the 30 μM used for covalent conjugation. IC_50_ values for **1** and **2** were found to drop by ∼2 fold on preincubation, possibly reflecting inhibition of non-covalently *versus* covalently modified PHD2.

The site of crosslinking of **3** was investigated using protein variants. First, **3** was incubated with C-terminal truncated PHD2 (PHD2_181–402_) and a loop deletion variant (deletion of residues 238–250, PHD2_Δ_). Crosslinking was maintained with the loop truncate, but near completely absent in C-terminally truncated PHD2, suggesting a Lys-residue (K408, K416 or K423) in the C-terminal region is involved in crosslinking (Fig. 3A). Thus, **3** was incubated with PHD2 constructs truncated at residues 402, 410, 414 and 418. Both the 418 (PHD2_181–418_) and 414 (PHD2_181–414_) truncated proteins were covalently modified by **3**, suggesting K423 and K416 are not involved in crosslinking (Fig. S10, ESI†). Crosslinking did not occur with the 410 truncate; however, there are no Lys-residues between 411 and 414, so it is possible K408 is involved and that the truncation at 410 alters the conformation such that crosslinking does not occur. Crosslinking experiments with PHD2 R411M implied that R411 is not directly involved in crosslinking (Fig. S11, ESI†).

**Fig. 3:**
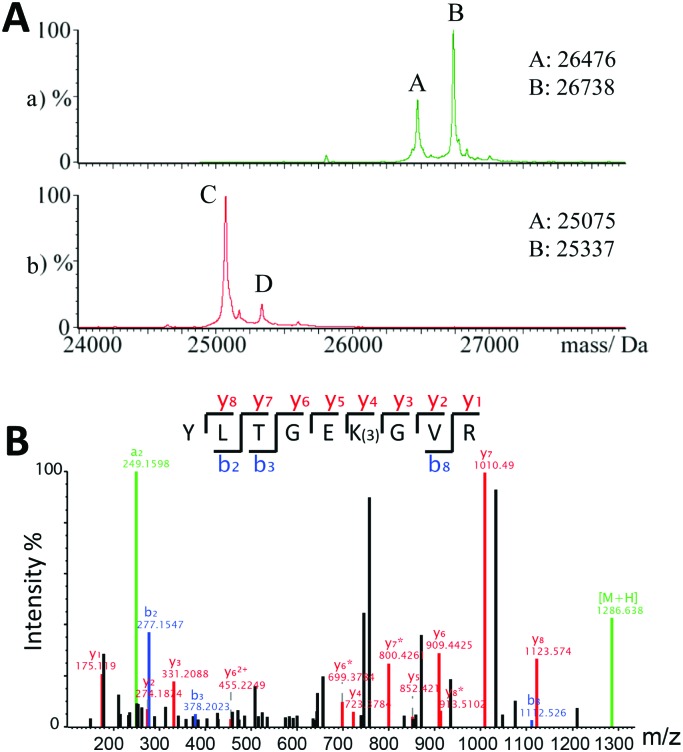
(A) Non-denaturing ESI-MS data on the flexible loop truncated (top) and C-terminal tail tuncated (bottom) versions of PHD2 in the presence of equimolar Fe(ii) and **3** (3 hour incubation, 37 °C. Cone voltage: 200 V). A = [PHD2_Δ_ + Fe], B = [PHD2_Δ_ + Fe + **3**], C = [PHD2_181–402_ + Fe], D = [PHD2_181–402_ + Fe + **3**]. (B) MS/MS spectrum of the trypsin derived peptide _403_YLTGEKGVR_411_ crosslinked to **3** identified K408 as the site of crosslinking.

The site of crosslinking was investigated by trypsin digest and LC-MS/MS. The imine adducts formed by **1**/**2** were fixed by reduction using sodium cyanoborohydride (Fig. S12, ESI†). Modified protein samples were digested (trypsin) and analysed by ‘nano LC-MS/MS’.^19^ In all 3 samples, modification by the respective inhibitor was identified on the trypsin-derived peptide _403_YLTGEKGVR_411_, and localised to K408 (Fig. 3B and Fig. S13, ESI†). This observation is supported by experiments with PHD2 K408I that did not undergo apparent covalent modification (<10%) with **3** (Fig. S11, ESI†). Consistent with these results, the human PHD isoforms PHD1 and PHD3, which possess Gly and Arg residues at the analogous position to K408 in PHD2, did not covalently react with **3** (Fig. S14, ESI†).

Cellular PHD inhibition in cells increases HIF-α levels, so mimicking the hypoxic response. The effects of **1**, **2** and **3** on HIF-1α regulation in HeLa cells were studied, but no upregulation of HIF-1α was observed (Fig. S15, ESI†), possibly reflecting poor permeability of these carboxylic acids. Ester derivatives **5** and **6** were synthesised and caused upregulation of HIF-1α (Fig. S16, ESI†). All tested doses caused a small upregulation of HIF-1α, which was apparently independent of tested concentrations, and much smaller than observed for **FG2216**. This HIF-1α induction may be due formation of covalently modified PHD2, which as described above is less active than WT PHD2 (covalent PHD2 modification of PHD2 may also promote its degradation). High concentrations of **5** (>250 μM) induced more pronounced HIF-1α upregulation, which may involve inhibition of (covalently modified) PHD2 by the free **1**, consistent with IC_50_ values for inhibition of isolated PHD2·Fe·**1** by **1** (Table S1, ESI†).

Ligands that covalently modify proteins are used for activity-based profiling by functionalisation with an appropriate handle.^20^ The observation that covalently modified PHD2 is active suggests an additional application, *i.e.* incorporation of a photoreactive group in the ligand to photocrosslink with substrates, which could then be isolated and identified. This method for incorporation of a photoreactive group near the active site of a protein to enable substrate capture is complementary to ‘photoreactive site directed mutagenesis’.^21^,^22^ Azide **7** was designed for use as a tag to allow ‘click’ conjugation with a handle (*e.g.* biotin) for activity-based profiling, or as a photoreactive aryl azide for substrate capture. **7** covalently modified PHD2 at a similar rate to **3** (Fig. 4). To explore the potential of **7** in protein profiling, biotinyl cyclooctyne (**8**) was added to the modified protein, causing near complete conversion of covalent PHD2·Fe·**7** to biotinylated PHD2·Fe·**7**.**8** (Fig. 4). The photoreactivity of the covalently modified protein was investigated by irradiation of ‘covalent’ PHD2·Fe·**7** with UV light. MS analysis indicated near complete conversion of the adduct to a species with a mass of 28 Da less, implying nitrogen loss and insertion of the resulting nitrene into a protein bond (Fig. 4). There is scope for optimisaiton and broader application of the approach described here. We found **7** inhibits a subclass of Fe/2OG histone demethylases, albeit to a lesser extent than the established inhibitors IOX1 and 2,4-pyridinedicarboxylic acid (Table S2, ESI†). Notably, we did not accrue evidence that the human histone demethylase KDM4A undergoes covalent reaction with **3** (Fig. S14, ESI†). Work toward the application of **7** and derivatives in protein profiling and substrate capture in cells is ongoing.

**Fig. 4:**
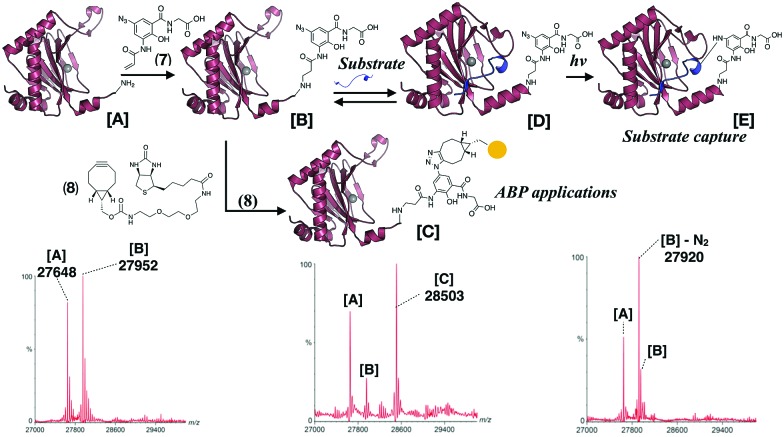
Schematic representation of proposed applications of azide **7** for activity-based protein profiling and substrate capture. Left: Modification of PHD2 by **7** after 2.5 h incubation, middle: subsequent strain promoted cycloaddition reaction enables conjugation of the protein with a biotinyl cyclooctyne **8**, right: UV irradiation of PHD2 + **7** leads to loss of nitrogen (N_2_), indicating modified protein is photoreactive.

Overall the results reveal the potential for the modulation (either inhibition or activation) of PHD2, and more generally 2OG oxygenases, by covalent reaction of an enzyme region involved in substrate binding. Azide functionalisation of one of the covalently reacting molecules enabled subsequent conjugation with biotin, offering application in activity-based protein profiling. This azide was also found to be photoreactive, suggesting a strategy for covalent capture of PHD2 substrates.

Our work was supported by the Newton-Abraham Fund (J. M.), the British Heart Foundation (R. K. L.), the Wellcome Trust (WT 106244/Z/14/Z) and Cancer Research UK (C8717/A18245).

## Conflicts of interest

There are no conflicts to declare.

## Supplementary Material

Supplementary information
