# Supplementary material for: Small-molecules that covalently react with a human prolyl hydroxylase – towards activity modulation and substrate capture
Source: Chem Commun (Camb). 2018 Nov 19;55(8):1020–3. doi: 10.1039/c8cc07706a (PMC6350621; doi:10.1039/c8cc07706a)
Supplement: Supplementary file 1 [file CC-055-C8CC07706A-s001.pdf]

Electronic Supplementary Material (ESI) for ChemComm.
This journal is © The Royal Society of Chemistry 2018

Supplementary Information

Small-molecules that covalently react with a human prolyl hydroxylase – Towards activity modulation and substrate capture

Jacob T. Bush,[a] Robert K. Leśniak,[a] Tzu-Lan Yeh,[a] Roman Belle,[b] Holger Kramer,[a] Anthony Tumber,[a] Rasheduzzaman Chowdhury,[a] Emily Flashman,[a] Jasmin Mecinović,[*abc] and Christopher J. Schofield[*a]

[a] Department of Chemistry, University of Oxford, 12 Mansfield Road, Oxford, OX1 3TA, United Kingdom.

[b] Institute for Molecules and Materials, Radboud University, Heyendaalseweg 135, 6525 AJ Nijmegen, The Netherlands.

[c.] Department of Physics, Chemistry and Pharmacy, University of Southern Denmark, Campusvej 55, 5230 Odense, Denmark.

1. Figures

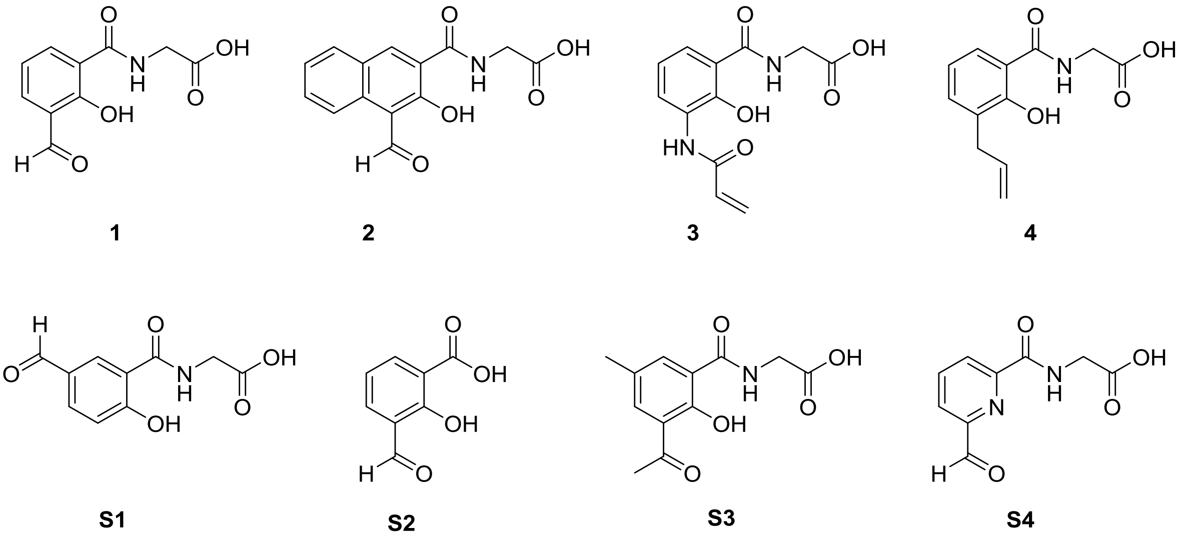

Fig. S1 Structures of glycine-coupled salicylic acids and analogues used in this work.

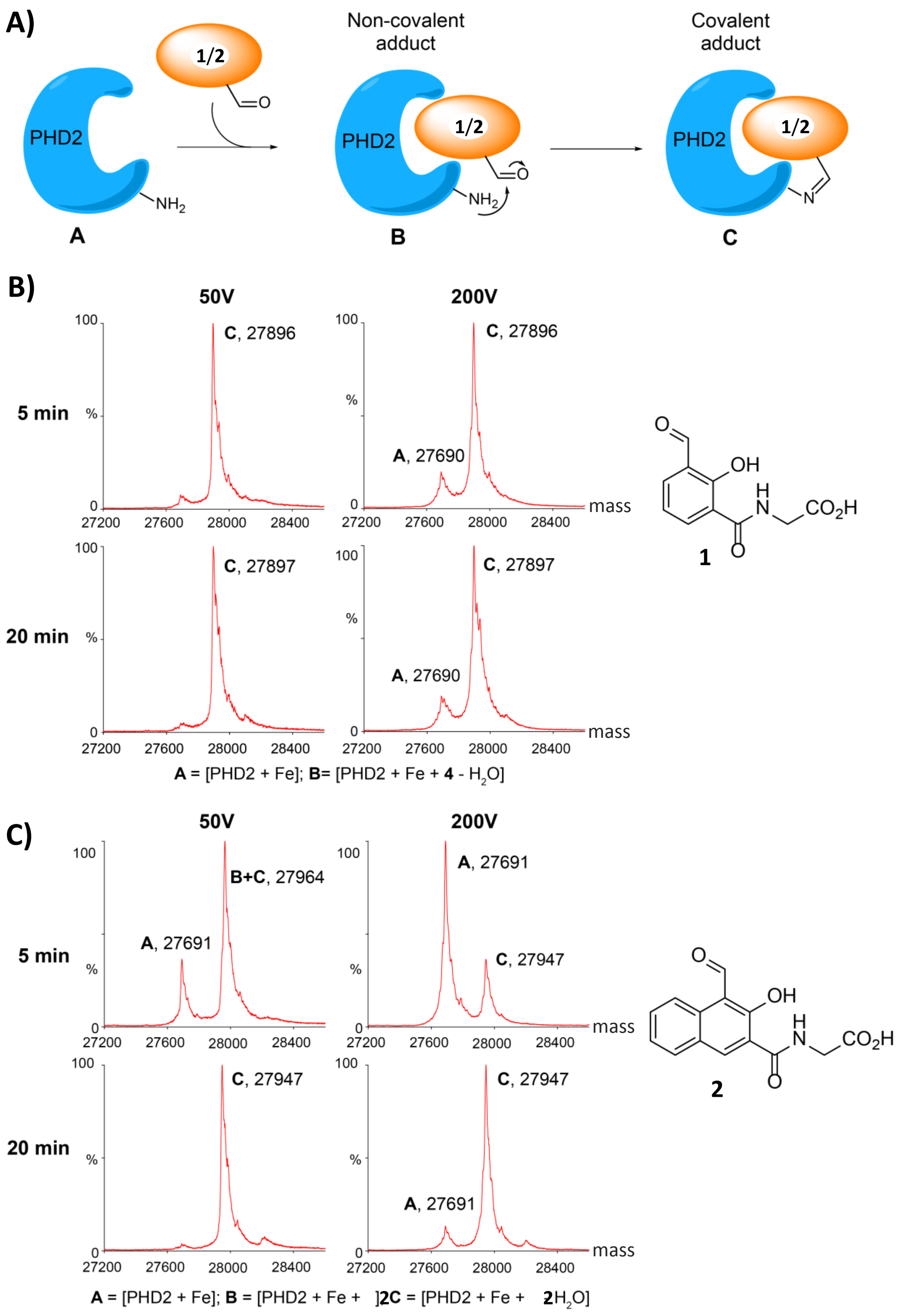

Fig. S2 A) Schematic representation of the assigned species formed on addition of **1** or **2** to PHD2. The results support initial non-covalent binding of the inhibitors to PHD2.Fe(II) to form **B**. Subsequently, condensation of a lysine residue with the aldehyde forms the covalent adduct **C**. (**B**) and **(C):** Non-denaturing ESI-MS data support the covalent crosslinking of **1**/**2** with PHD2. Conditions: PHD2 (20 μM) was incubated (37 °C, 5 or 20 min) with equimolar $FeSO_4$ and **1** or **2**, and the resulting mixtures were analysed by ESI-MS (cone voltage: 50 V or 200 V). Labelled peaks correspond to: **A**: Unmodified PHD2.Fe. **B**: Non-covalent adduct PHD2.Fe.**2**. **C**: Covalent adduct PHD2.Fe.**1**/**2**.

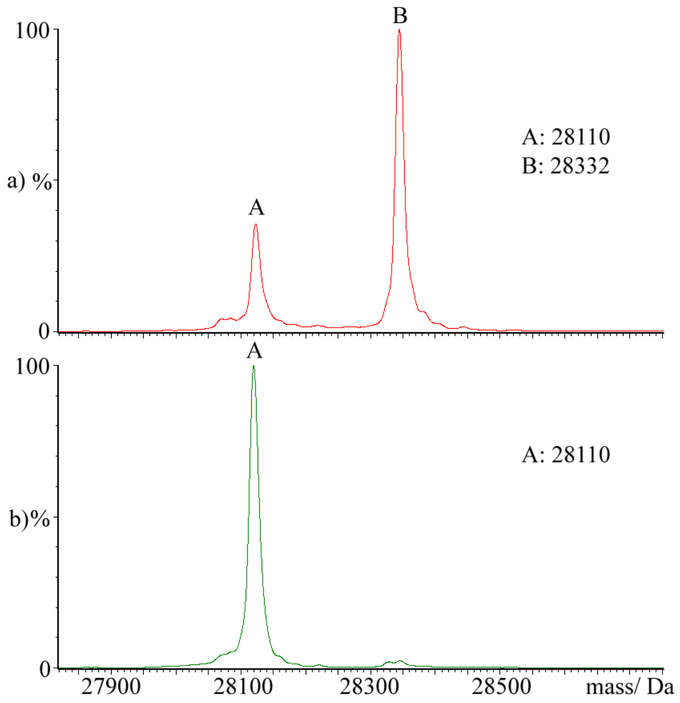

Fig. S3 Non-denaturing ESI-MS analysis of PHD2 in the presence of equimolar amounts of $FeSO_4$ and aldehyde **S1** after 20 minutes incubation at 37 ºC in ammonium acetate (15 mM, pH 7.5) at sample cone voltages (a) 80 V or (b) 200 V. Peaks observed correspond to: (A) the PHD2.Fe complex; (B): the non-covalent PHD2.Fe.**S1** complex.

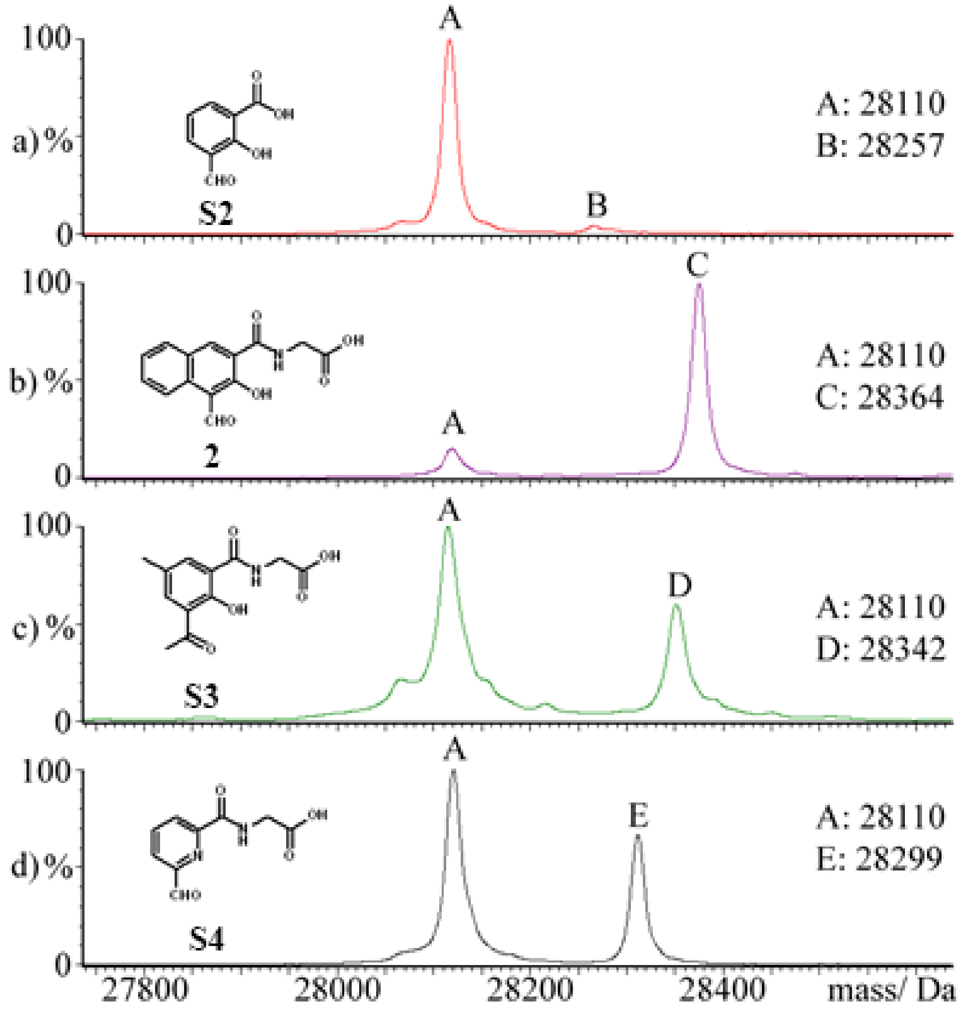

Fig. S4 Non-denaturing ESI-MS analysis of PHD2 in the presence of equimolar amounts of $FeSO_4$ and (a) **S2**; (b) **2**; (c) **S3**; (d) **S4** after 20 minutes incubation at 37ºC in ammonium acetate (15 mM, pH 7.5) at a sample cone voltage 200V. Peaks observed are consistent with formation of the following complexes: (A): PHD2.Fe; (B): covalently linked PHD2.Fe.**S2** complex; (C): covalently linked PHD2.Fe.**2** complex; (D): covalently linked PHD2.Fe.**S3** complex; (E): covalently linked PHD2.Fe.**S4** complex.

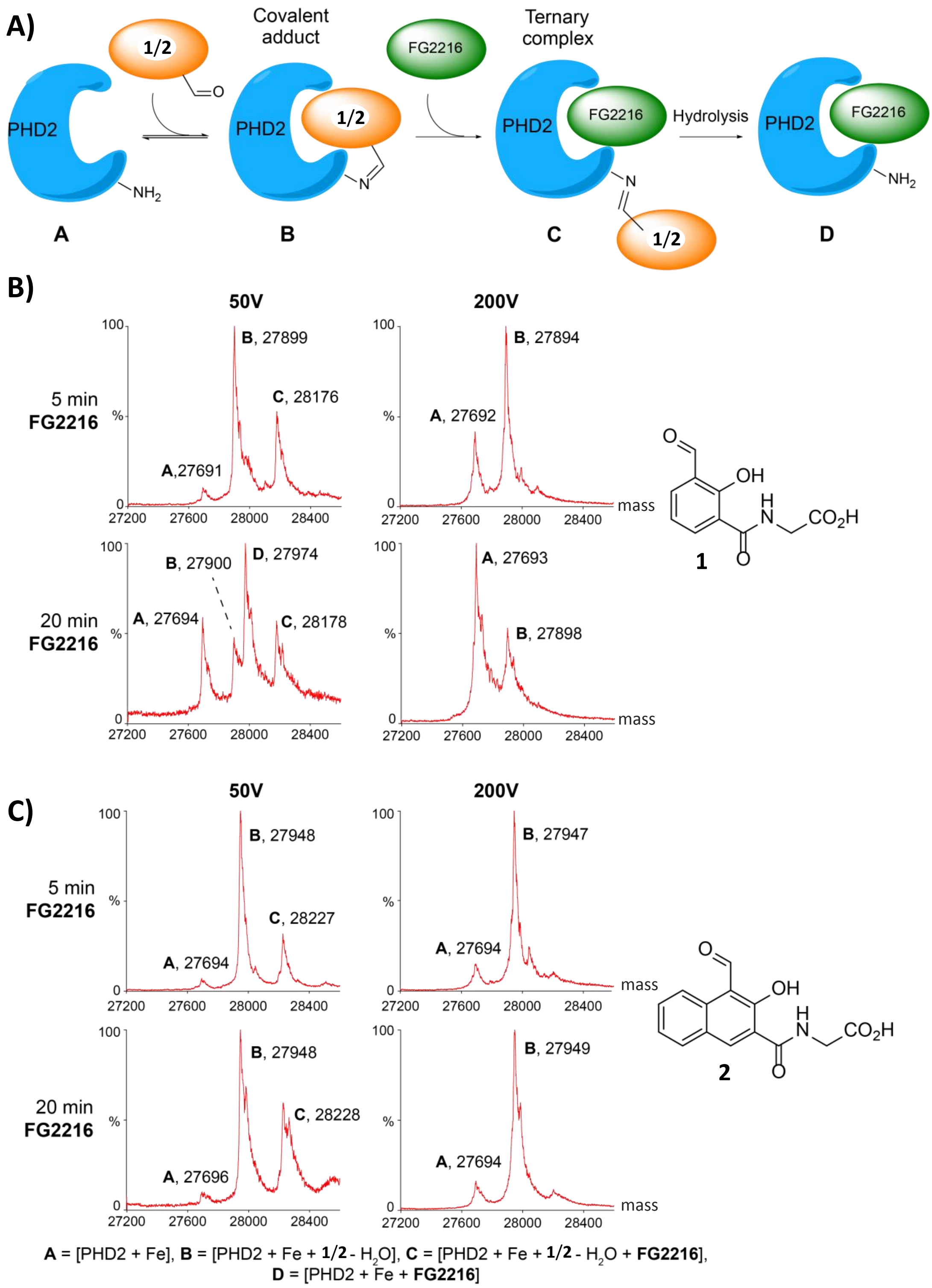

Fig. S5 Evidence the inhibitor **FG2216** can bind to PHD2 that is covalently modified by **1/2**. PHD2 was incubated (37 °C, 20 min) with equimolar $FeSO_4$ and aldehyde (**1** or **2**) to enable formation of the covalent adduct. Subsequently, **FG2216** (20 µM) was added, followed by further incubation (37 °C, 5 or 20 min). The resultant mixtures were analysed at cone voltages of 50 V or 200 V. (**A)** Schematic representation of the possible species formed upon addition of **1** or **2** to PHD2 followed by **FG2216** addition. **(B)** and **(C):** Non-denaturing ESI-MS analyses indicate that **FG2216** can bind to PHD2 that is covalently modified by **1** or **2**.

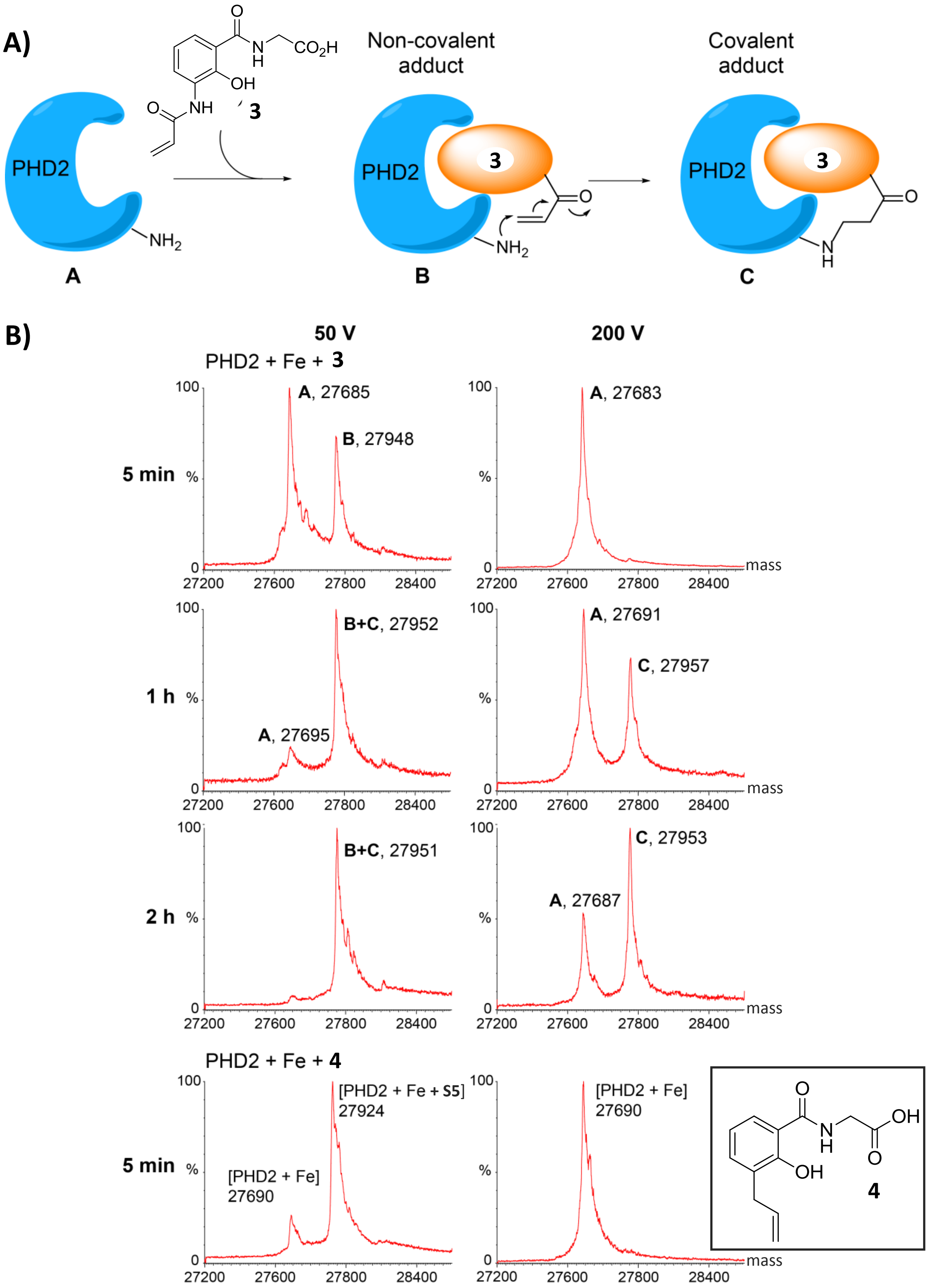

Fig. S6 The α,β-unsaturated amide **3** can covalently modify PHD2. (**A**) Schematic representation of the adducts formed upon incubation of **3** with PHD2. (**B**) Non-denaturing ESI-MS data demonstrating that **3** can covalently crosslink with PHD2. PHD2 (20 µM) was incubated (37 °C, 5 min, 1 h or 2 h) with equimolar $FeSO_4$ and **3** or **4** before analysis by ESI-MS (cone voltage: 50 V or 200 V). Labelled peaks correspond to the following assigned complexes: **A**: unmodified PHD2.Fe; **B**: the non-covalent PHD2.Fe.**3** adduct; **C**: the covalent PHD2.Fe.**3** adduct.

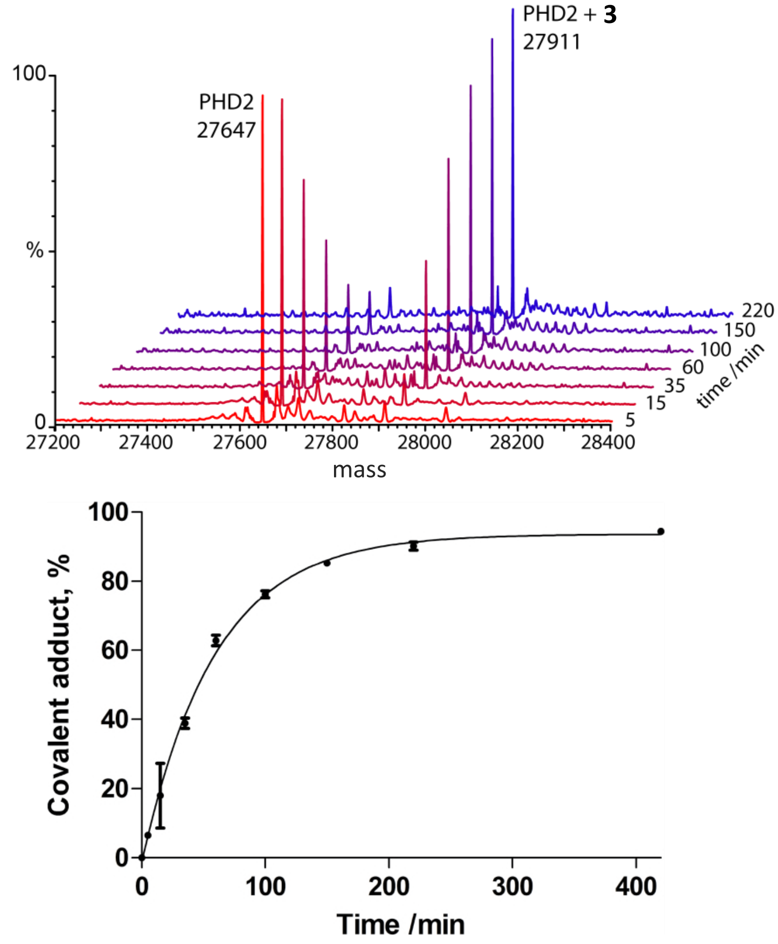

Fig. S7 Time-course analysis of crosslinking between PHD2 and **3**. PHD2 (20 µM) was incubated (37 °C, 4 h) with $FeSO_4$ (20 µM) and **3** (30 µM). Samples were taken at regular intervals, quenched by acidification and the ratio of covalent crosslinking analysed by ESI-MS under denaturing conditions. **Above**: overlaid deconvoluted mass spectra; **Below**: variation of crosslinking with time. Data represent mean intensities of repeats (n=3), error bars show 1 standard deviation.

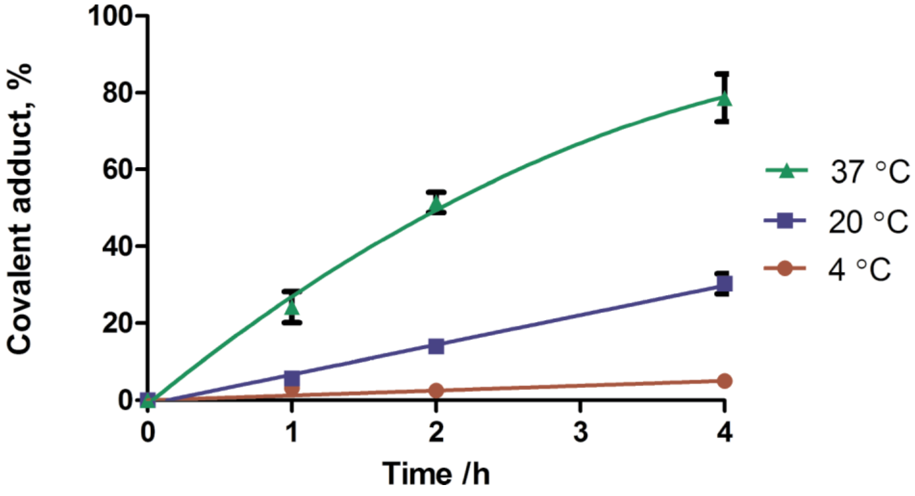

Fig. S8 Temperature dependence of the rate of crosslinking of **3** with PHD2. PHD2 (20 µM) was incubated with equimolar Fe(II) and **3** in buffer (50 mM Tris, 150 mM NaCl, pH 7.5) at 4, 20 and 37 °C. The ratio of crosslinking after 1, 2, and 4 h was determined by ESI-MS under denaturing conditions (n = 2), error bars represent 1 standard deviation.

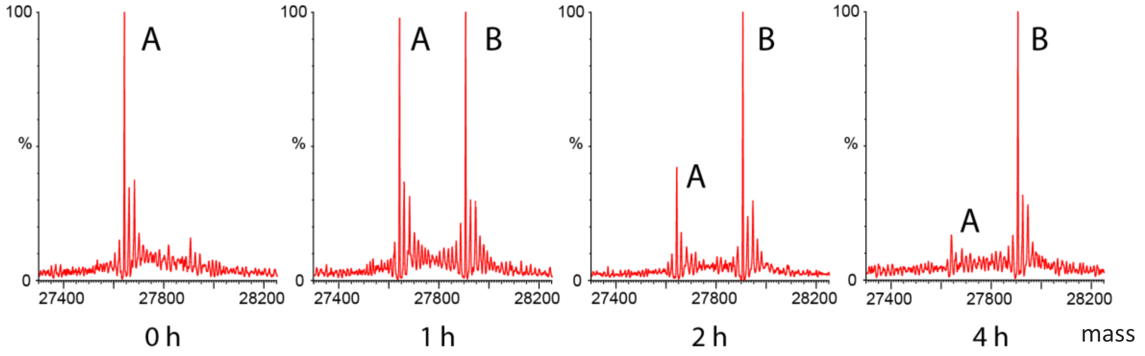

Fig. S9 Ratio of crosslinked PHD2.**3** before evaluation of catalytic activity. Labelled peaks correspond to: (**A**): PHD2, (**B**): PHD2.**3**.

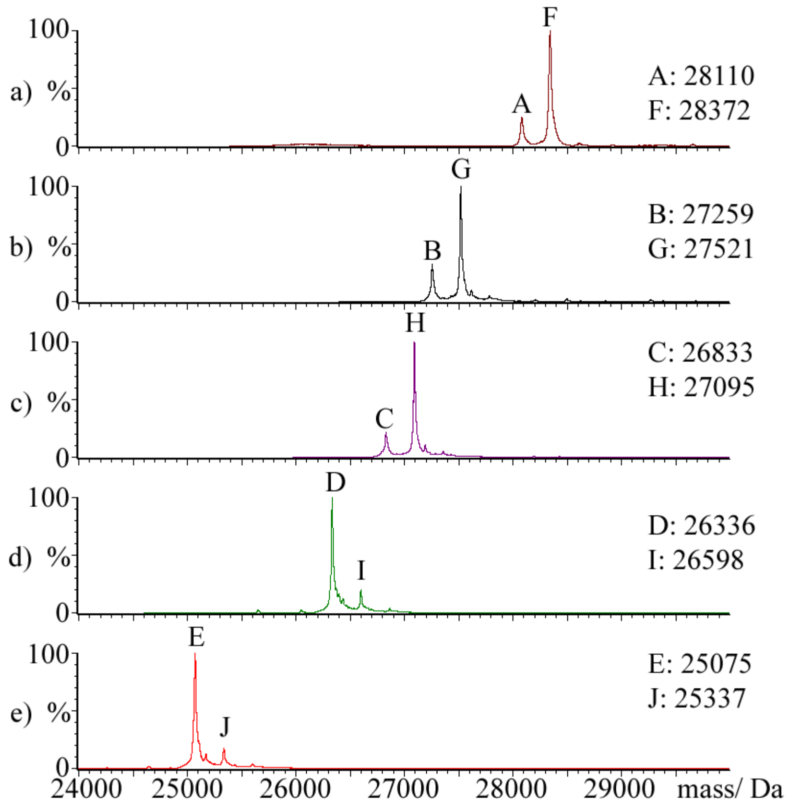

Fig. S10 Non-denaturing ESI-MS analysis of (a) PHD2 and its *C*-terminal truncations (b) $PHD2_{181\text{-}418}$; (c) $PHD2_{181\text{-}414}$; (d) $PHD2_{181\text{-}410}$; (e) $PHD2_{181\text{-}402}$ in the presence of equimolar amounts of $FeSO_4$ and **3** after 3 hours incubation at 37ºC in ammonium acetate (15 mM, pH 7.5) at a sample cone voltage of 200V. Peaks present: (A): PHD2.Fe; (B): $PHD2_{181\text{-}}$

$_{418}$.Fe; (C): PHD2$_{181\text{-}414}$.Fe; (D): PHD2$_{181\text{-}410}$.Fe; (E): PHD2$_{181\text{-}402}$.Fe; (F): PHD2.Fe.**3**; (G): PHD2$_{181\text{-}418}$.Fe.**3**; (H): PHD2$_{181\text{-}414}$.Fe.**3**; (I): PHD2$_{181\text{-}410}$.Fe.**3**; (J): PHD2$_{181\text{-}402}$.Fe.**3**.

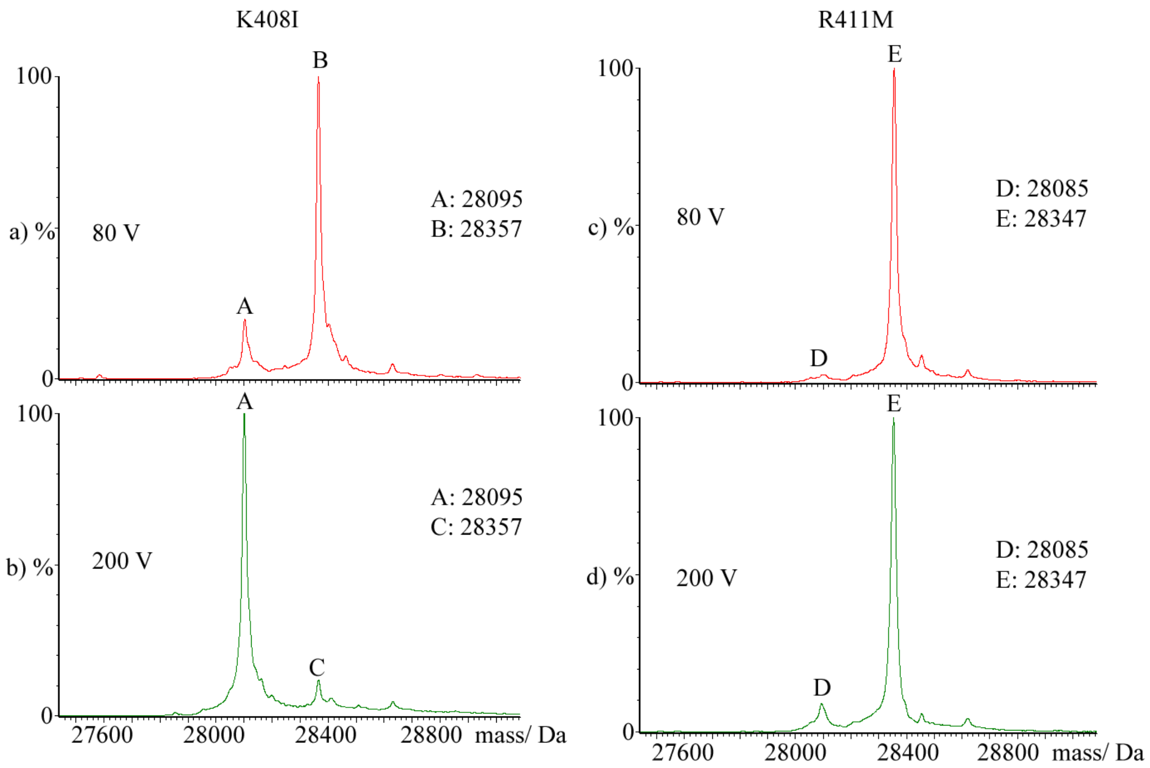

Fig. S11 Non-denaturing ESI-MS data of K408I PHD2 in the presence of equimolar amounts of $FeSO_4$ and the Michael acceptor **3** after 3 hours incubation at 37ºC in ammonium acetate (15 mM, pH 7.5) at a sample cone voltages of (a) 80V or (b) 200V. Non-denaturing ESI-MS data of R408M PHD2 in the presence of equimolar amounts of $FeSO_4$ and **3** after 3 hours incubation at 37ºC in ammonium acetate (15 mM, pH 7.5) at a sample cone voltage of (c) 80V or (d) 200V. The peaks observed are consistent with the following complexes: (A): K408I.Fe; (B): non-covalent K408I.Fe.**3** complex, (C): covalent K408I.Fe.**3** complex; (D): R411M.Fe; (E): covalent R411M.Fe.**3** complex.

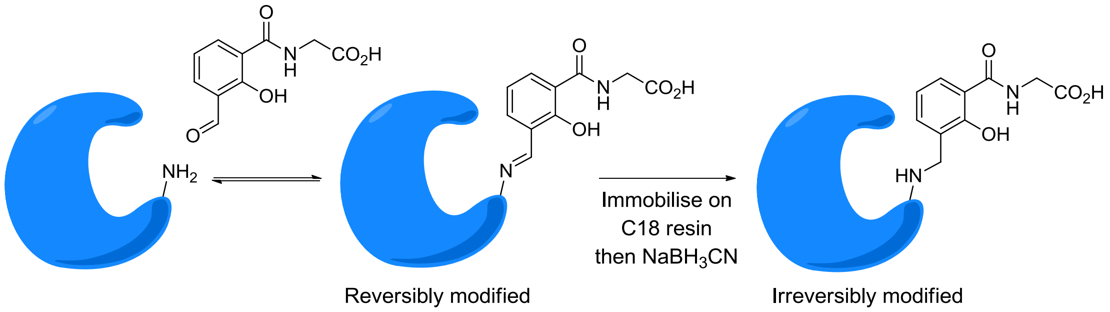

Fig. S12 Reduction of PHD2.**1/2** imine adducts. PHD2 (20 µM) was incubated (1 h, 37 °C) with equimolar Fe(II) and aldehyde **1** (or **2**) in buffer (15 mM ammonium acetate, pH 7.5) to enable formation of the imine adduct. The modified enzyme was subsequently immobilised on a C18 column, and the imine reduced by eluting (5 mL, 1 mL min^{-1}) with $NaBH_3CN$ (30 mM) in buffer (50 mM sodium phosphate, pH 7.5).

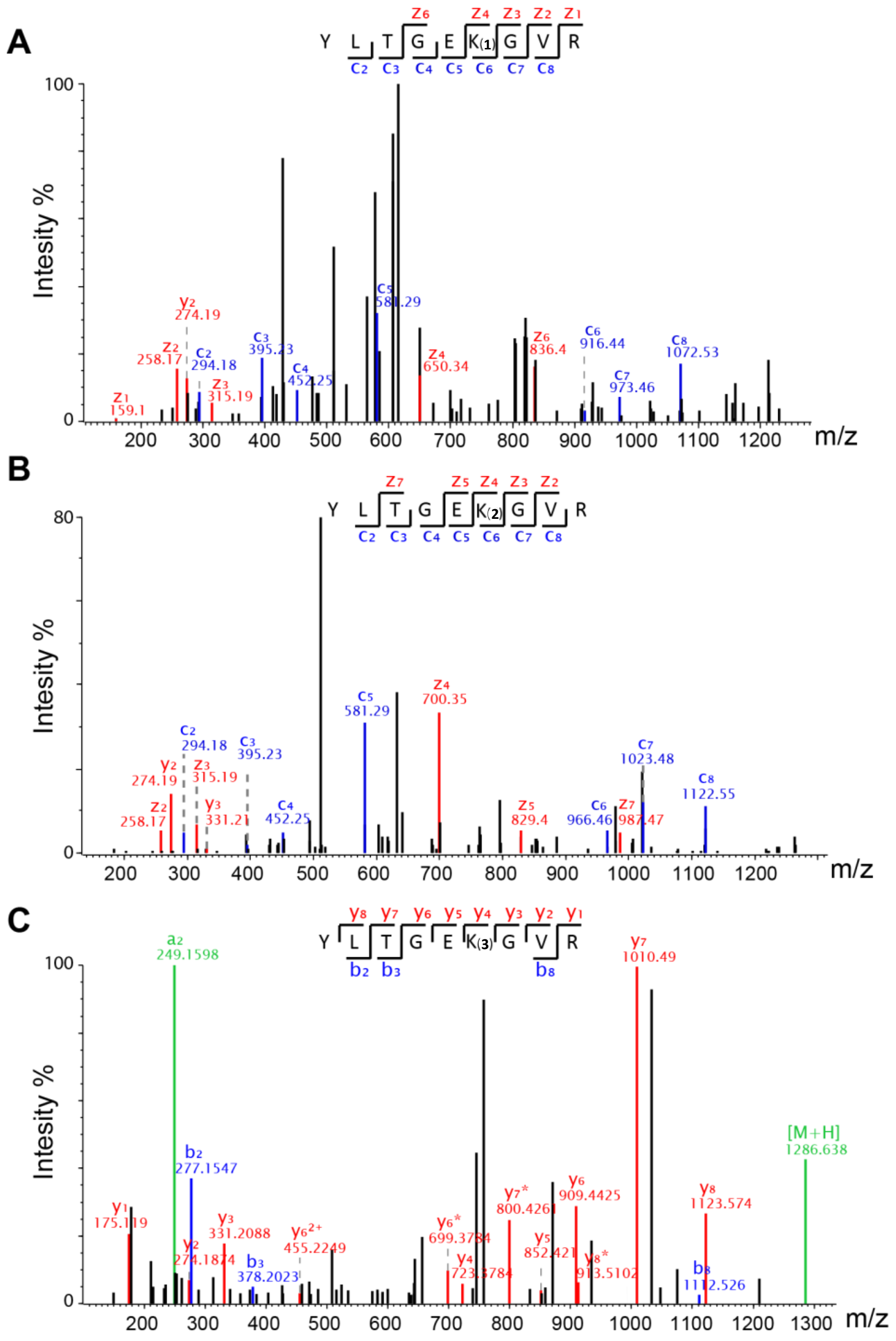

Fig. S13 MS/MS assignment of K408 as the site of crosslinking of **1** (**A**), **2** (**B**) and **3** (**C**) with PHD2. Crosslinked samples [PHD2-**4**/**5**/**6**] were digested using trypsin and analysed by LC-MS/MS. Processing of the data (MaxQuant) implied all of the small molecules had reacted with K408. Highest scoring MS/MS spectra of the trypsin derived peptide $_{403}$YLTGEKGVR$_{411}$ crosslinked to **1** (**A**), **2** (**B**) and **3** (**C**) are shown. **A**: Peptide + **1** – H_2O + 2H, [M + 3H]$^{3+}$, m/z 410.544 Da (MW = 1228.61 Da), fragmentation by electron transfer dissociation (ETD). **B**: Peptide + **2** – H_2O + 2H, [M + 2H]$^{2+}$, 974.939 Da (MW = 1947.86), fragmentation by ETD. **C**: Peptide + **3**, [M + 2H]$^{2+}$, m/z 643.822 Da, (MW = 1285.63 Da), fragmentation by higher-energy collisional dissociation (HCD). Ions resulting from a, b, c, y and z fragmentation are labelled. Fragmentation of the ArHN-C(O)CH_2 amide bond in the inhibitor **3** represented by *.

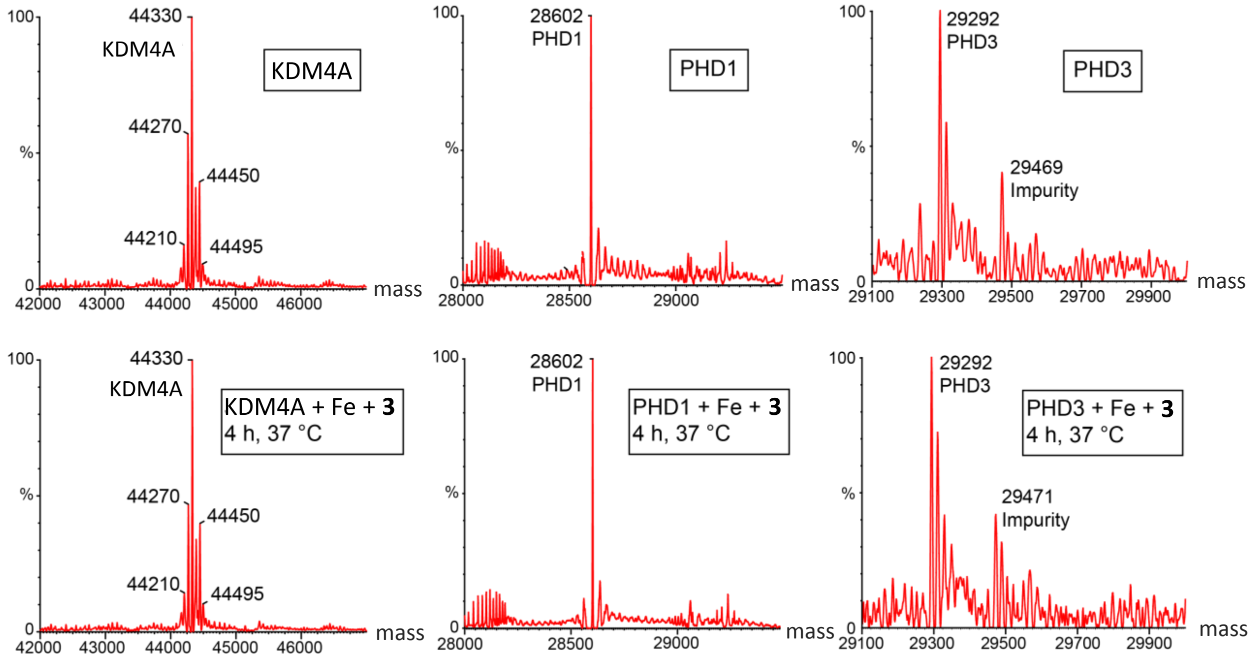

Fig. S14 Denaturing MS experiments showing a lack of covalent adducts between **3** and KDM4A, PHD1 and PHD3. The enzyme (20 µM), Fe(II) (30 µM) and **3** (30 µM) were incubated (37 °C for 4 h) and analysed by ESI-MS.

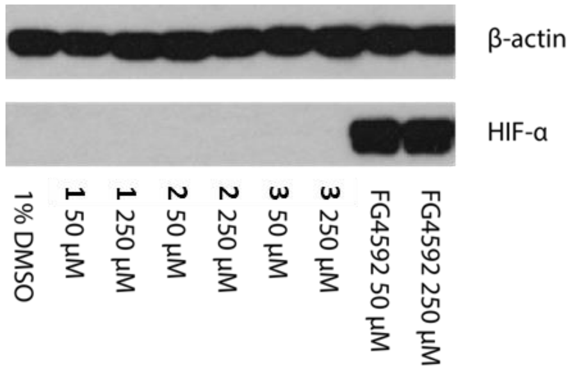

Fig. S15 HIF-1α response on treatment of live cells with covalent inhibitors **1**, **2** and **3**. HeLa cells were treated with the indicated covalently reacting inhibitors (50 or 250 µM, 6 h) before cell lysis and analysis of HIF-1α levels by anti-HIF-1α immunoblotting. The potent and cell permeable PHD2 inhibitor **FG4592** was used as a positive control.

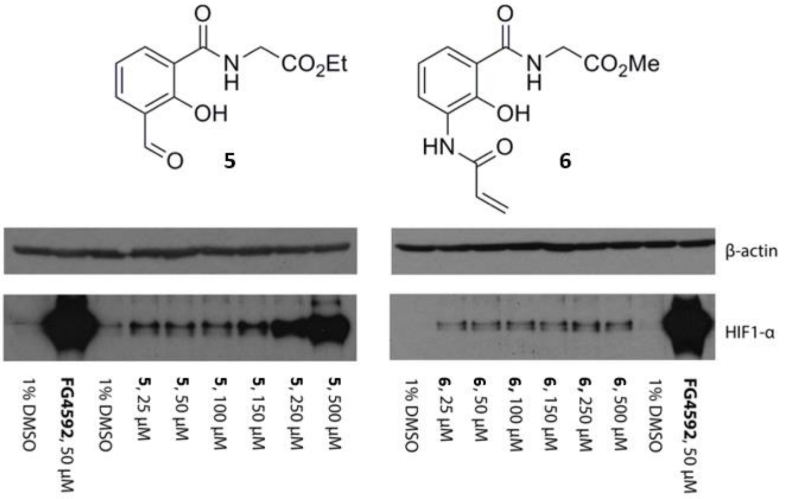

Fig. S16 Concentration dependence of the HIF-1α response to esters **5** and **6**. HeLa cells were treated with a range of concentrations of inhibitor (0-500 µM, 6 h) before cell lysis and analysis of HIF-1α levels by anti-HIF-1α immunoblotting. The inhibitor **FG4592** was used as a control.

Table S1. The potency (IC_{50}) of inhibitors **1-3** as determined by AlphaScreen™ assay after pre-incubation of PHD2, $FeSO_4$ and inhibitor. Experiments were carried out in duplicate; the errors are ± 1 standard deviation.

Probe	**1**		**2**		**3**
Pre-inc	2 min	20 min	2 min	20 min	4 h
IC_{50} (µM)	540±77	247±36	166±39	101±27	>2 mM

Table S2. The potency (IC_{50}) of **7** along with two control inhibitors as determined by AlphaScreen™ assay.

	IOX1	2,4-PDCA	**7**
KDM4A	5.2	2.4	18.2
KDM4D	9.1	5.3	10.3
KDM5B		1.8	13.6
KDM6B	0.37		8.4

2. Experimental procedures

1.1.1 Non-denaturing mass spectrometry[1]

Apo PHD2 was desalted using a Bio-Spin 6 Column (Bio-Rad, Hemel Hempstead, UK) into 15 mM ammonium acetate (pH 7.5). The stock solution was diluted with the same buffer to a final concentration of 100 μM. $FeSO_4 \cdot 7H_2O$ was dissolved in 20 mM HCl at a concentration of 100 mM. This solution was then diluted with MilliQ purified water to give a final working concentration of 100 μM. The protein was mixed with the Fe(II) solution and the inhibitor solution to give final concentrations of 20 μM PHD2, 20 μM Fe(II), and 20 μM inhibitor. The resultant solution was incubated for the appropriate time (specified in the text) at 37 °C. In competition experiments, **FG2216** was subsequently added and the resulting solutions further incubated (5 min or 20 min) at 37 °C. After incubation, the samples were immediately analysed by ESI-MS. Data were acquired using a Q-TOF mass spectrometer (Q-TOF micro, Micromass, Altrincham, UK) interfaced with a Nanomate (Advion Biosciences, Ithaca, NY, USA) with a chip voltage of 1.70 kV and a delivery pressure 0.5 psi (1 psi = 6.81 kPa). The sample cone voltage was typically 200 or 50 V with a source temperature of 40 °C and with an acquisition/scan time of 10s/1s. Sample acquisition was performed in positive ion mode in the range of 2000-4000 *m/z*. The pressure at the interface between the atmospheric source and the high vacuum region was fixed at 6.30 mbar. External instrument calibration was achieved using sodium iodide. Data were processed with MassLynx™ v4.0 (Waters) and protein peaks were deconvoluted using the Maximum Entropy method (MaxEnt1).

1.1.2 Denaturing mass spectrometry

Intact protein masses were obtaine by liquid chromatography-mass spectrometry (LC-MS) using a Waters LCT premier XE time-of-flight mass spectrometer interfaced with a Waters 1525u HPLC pump and Waters 2777 autosampler.

The protein sample (10 µM, 5 µl) was injected onto a Chromolith FastGradient RP-18 2x50mm Monolithic reverse phase HPLC column and eluted at 0.4 $ml.min^{-1}$ using a gradient system from Solvent A (water, 0.1 % (v/v) formic acid) to Solvent B (acetonitrile, 0.1 % (v/v) formic acid) according to the following conditions:

Time (Min)	%A	%B	Flow Rate ($ml.min^{-1}$)
0:00	95	5	0.4
1:00	95	5	0.4
5:00	0	100	0.4
8:00	0	100	1
8:10	95	5	1
9:30	95	5	1

The eluent was transferred directly to the mass spectrometer. The following MS parameters were used: Capillary voltage – 3,000 V; Sample cone voltage – 50 V; Extraction cone voltage – 1V; Desolvation temperature – 250°C; Source temperature – 100°C; Cone gas flow – 100 $l.hour^{-1}$; Desolvation gas flow (N_2) – 830 $l.hour^{-1}$. Data were processed with MassLynx™ v4.0 (Waters) and protein peaks were deconvoluted using the Maximum Entropy method (MaxEnt1).

1.1.3 Analysis of protein activity by peptide turnover assay using MALDI-MS detection[2]

PHD2 (20 µM), Fe(II) (20 µM) and the inhibitor (30 µM) in buffer (15 mM ammonium acetate, pH 7.5) were incubated for the appropriate time (specified in the text) at 37 °C. Subsequently, the enzyme (1.5 µL, final concentration 1 µM) was added to a mixture containing HIF-1α CODD peptide (50 µM, HIF-$1\alpha_{556\text{-}574}$), ascorbate (1 mM), 2-oxoglutarate (100 µM) and the respective inhibitor (30 µM) in buffer (50 mM Tris, 150 mM NaCl). In the positive control no inhibitor was added. The resultant mixture was incubated at 37 °C and samples (3 µM) were taken at regular intervals and quenched by addition of 1% (v/v) aqueous formic acid (3 µM). Samples were analysed using a MALDI micro MX mass spectrometer (Waters) in the reflectron positive ion mode. Data were processed using MassLynx v4.0. The yield of crosslinking was determined by comparison of the signal intensities for crosslinked and unmodified protein.

1.1.4 Analysis of protein activity by peptide turnover assay using the AlphaScreen™ [3]

The assay consisted of three parts: pre-incubation with inhibitor, the PHD2 hydroxylation reaction, and incubation with the AlphaScreen™ bead mix. The assay was carried out in 384-well white ProxiPlates (PerkinElmer). The reaction was performed in buffer (50 mM HEPES, pH 7.5, 0.01% Tween-20 and 0.1% BSA) in a final volume of 10 µL at room temperature. The enzyme mix (5 nM PHD2 (residues 181-426), 20 µM Fe(II) and 200 µM ascorbate) was incubated with the inhibitor (1 min, 20 min or 1 h) prior to addition to the peptide mix (60 nM biotinylated CODD peptide-HIF-$1\alpha_{556\text{-}574}$ and 2 µM 2OG). The resulting solution was incubated for 10 min at room temperature before the hydroxylation reaction was quenched by addition of EDTA (5 µL, 30 mM). 5 µL of pre-incubated donor-acceptor bead mix (AlphaScreen streptavidin-conjugated donor and ProteinA-conjugated acceptor beads; PerkinElmer) with HIF-1α hydroxy-Pro546 antibody (Cell Signalling) was added to the reaction mixture followed by incubation for 1 h in the dark at room temperature. The luminescence signal was acquired with an Envision (Perkin Elmer) plate reader.

1.1.5 LC-MS/MS analysis of the site of crosslinking

Reduction of the imine adducts formed by the aldehyde inhibitors[4]

A SepPak cartridge (Waters) was washed with acetonitrile (2 mL) and then conditioned with solvent A (2 mL). The protein was loaded onto the SepPak cartridge and the cartridge was then flushed with reducing reagent (5 mL, 30 mM sodium cyanoborohydride in 50 mM sodium phosphate buffer, pH 7.5) over a period

of 10 min. The labelling reagent was removed by flushing with solvent A (2 mL), and the protein was eluted with solvent B (1 mL). The solvent was removed by vacuum centrifuge, and the protein reconstituted in 8 M urea prior to digestion using trypsin.

Solvent A – 0.6% acetic acid; Solvent B – 0.6% acetic acid and 80% acetonitrile.

Protein digestion and LC-MS/MS[5]

Protein samples (50 µL) were denatured by addition of 8 M urea (150 µl) and reduced with DTT (2 µl, 200 mM) at 56 °C for 25 min. The samples were cooled to room temperature and the cysteinyl residues were alkylated by addition of iodoacetamide (4 µl, 36 mg/ml) and incubation in the dark (20 min, room temperature). Dithiothreoitol (2 µl, 200 mM) was added to neutralise the iodoacetamide, and the samples were diluted with buffer (600 µl, 50 mM ammonium bicarbonate), before addition of trypsin (1:50, w/w). The resulting mixture was incubated for 16 h at 37°C and then quenched by addition of formic acid (10 uL).

Digests were analysed using an Orbitrap Elite™ machine (Thermo Fisher Scientific™, DE) connected to a UPLC Proxeon EASY-nLC 1000 and an EASY-Spray nano-electrospray ion source. Peptides were trapped on an Acclaim PepMap® trapping column (100 µm i.d. x 20 mm, 5 µm C18) and separated on an EASY-spray Acclaim PepMap® analytical column (75 µm i.d. x 500 mm, RSLC C18, 2 µm, 100 Å). Solvent A consisted of 0.1% (v/v) formic acid and solvent B of 0.1% (v/v) formic acid in acetonitrile. Peptides were separated using a gradient of 7% to 30% (v/v) solvent B at a flow rate of 200 nL/min. Gradient lengths are specified in the text and were either 30 min, 1 h, 2 h, 3 h or 4 h. Full scan MS spectra were acquired using the Orbitrap machine (350-1500 m/z, resolution 120,000, AGC target 1e6, maximum injection time 250 ms). CID and ETD spectra were acquired in the Ion Trap (resolution 7500, AGC cation target 3e4, AGC Anion target 2e5, maximum injection time 100 ms). The normalised collision energy for CID and HCD was set to 35% or 32 %, respectively. The 20 most intense peaks in the full MS scan were selected for fragmentation using either HCD, CID, or using a data-dependent decision tree (DDDT) to select between CID and ETD. In DDDT mode, ETD fragmentation was used for charge states 3, 4 and 5 with m/z less than 750 and for all charge states greater than 5. CID fragmentation was used for all other peptides. Dynamic exclusion was enabled (exclusion list size 75, exclusion duration 5 s).

The raw data files generated were processed using MaxQuant software (Version 1.4.1.2), integrated with the Andromeda search engine as described elsewhere. Data were searched against PHD2 protein sequence, as well as list of common contaminants defined by Andromeda. Protein and PSM false discovery rates (FDR) were set at 0.01. The minimum number of quantified peptides required per protein was set to 2. The maximum number of missed cleavages was set to be 2. Oxidation (M) and acetylation (*N*-term) were allowed as a variable modifications and carbamidomethylation (C) was used as a fixed modification. The covalent inhibitor modifications were created in Andromeda and allowed to occur on any nucleophilic residue. All assignments of modifications were validated by manual inspection of spectra.

1.1.6 Photoreaction studies

PHD2 (20 µM), Fe(II) (20 µM) and **6** (20 µM) in buffer (15 mM ammonium acetate, pH 7.5) were incubated for 4 h at 37 °C. The sample was cooled to 4 °C before irradiation with UV light (12 min, 310 nm, 4 °C) using a CaproBox™. Products were analysed by denaturing liquid chromatography-mass spectrometry. Data were processed with MassLynx™ v4.0 (Waters) and protein peaks were deconvoluted using the Maximum Entropy method (MaxEnt1).

1.1.7 Strain promoted 'click' reaction

PHD2 (20 µM), Fe(II) (20 µM) and **6** (20 µM) in buffer (15 mM ammonium acetate, pH 7.5) were incubated for 4 h at 37 °C. Bicyclo[6.1.0]non-4-yn-9-ylmethyl (2-(2-(2-(6-oxo-6-(2-oxohexahydro-1H-thieno[3,4-d]imidazol-4-yl)hexanamido)ethoxy)ethoxy)ethyl)carbamate **7** (100 µM) was added and the resultant solution incubated for 1 h at 37 °C. Products were analysed by denaturing liquid chromatography-mass spectrometry. Data were processed with MassLynx™ v4.0 (Waters) and protein peaks were deconvoluted using the Maximum Entropy method (MaxEnt1).

1.1.8 Preparation of PHD2 and variants

1.1.8.1 Site-directed mutagenesis[6]

Stratagene's QuikChange site-directed mutagenesis kit (Stratagene Instruction Manual #200518) was used to generate point mutations. Forward and reverse primers, each incorporating the mutation and complementary to opposite strands of the vector were used to amplify the sequence in opposite directions by PCR. A typical PCR was set up according to Stratagene's QuikChange® Site-Directed Mutagenesis protocol:

dsDNA template (0.1-0.5 ng/µL)	1 µL
forward primer (10 µM)	2 µL
reverse primer (10 µM)	2 µL
10× reaction buffer	5 µL
dNTP mixture (2 mM)	5 µL
sterile deionised H_2O	34 µL
Pfu Turbo DNA polymerase (2.5 U/µL)	1 µL

The PCR mixture was heated to 95ºC for 30 seconds to denature the DNA. Further thermal cycling (18 cycles) with the mutagenic primers yielded an amplified and mutated plasmid. Typical cycling parameters for QuikChange Site-Directed Mutagenesis are as follows:

Temperature	*Time*	*Step*
95ºC	30 seconds	Denaturation
57ºC	1 minute	Annealing
68ºC	1 minute/kb of plasmid length	Extension

The PCR reactions yielded product mixtures containing mutated plasmids and parental (non-mutated) dsDNA templates. It was necessary to remove the non-modified templates before transformation since they both contain the same antibiotic resistance gene. This was achieved by digesting the PCR products with the *Dpn*I restriction enzyme (Stratagene Instruction Manual #200518). Since *Dpn*I works on methylated/hemimethylated DNAs, the plasmid DNA templates were isolated from dam$^+$ *E. coli* strain so that the parent DNA could be digested. *Dpn*I (1 μL) was added to the PCR products, gently mixed and centrifuged for 1 minute before being digested for 3 hours at 37ºC. For tranformation, an aliquot of XL10-Gold competent cells (50 μL) was transferrd to a 1.5 mL sterile Eppendorf vial and the plasmid/PCR product (1 μL) was added with gentle mixing. Cells were treated with β-mercaptoethanol (2 μL) to enhance transformation efficiency. The transformation mixture was chilled on ice for 30 minutes, followed by a heat shock for 30 seconds at 42ºC to induce DNA uptake. The cells were incubated at 37ºC for one hour with 200 μl 2× TY medium and placed on LB-agar plates containing kanamycin (30 μg/mL). Finally, the plates were incubated at 37ºC. A single colony resulting from transformation was inoculated into 10 ml 2× TY medium containing kanamycin. The starter culture was grown overnight at 37ºC with shaking at 220 rpm. Plasmid DNA was purified from the culture using the Qiagen Miniprep Kit following the manufacturer's protocol. All plasmids were verified by DNA sequencing at the Geneservice Ltd., Department of Biochemistry, University of Oxford. The results were compared to wildtype sequence to ensure the amplified insert or the mutated plasmid sequence was as desired and in the correct reading frame.

1.1.8.2 Recombinant protein production[6]

A single colony was inoculated into 100 mL 2× TY media (overnight, 37ºC, shaken at 200 rpm) containing kanamycin (30 μg/mL). Then 7 ml of overnight starter culture was used to inoculate 2 L flasks containing 600 mL of 2× TY growth media with kanamycin (30 μg/mL). The flasks were incubated at 37ºC until the A_{600}

reached the range of 0.6-0.8, after which IPTG (600 μL, 0.5 mM) was added and growth was continued for an optimum incubation period (3-4 hours). Cells were harvested by centrifugation (9,000 rpm, 4ºC, 15 minutes) and the wet pellets were stored at -80ºC for protein purification. The frozen cell pellets were resuspended in 5 mL lysis buffer (see below) per gram of wet cell pellet. The cell suspension was then lysed by sonication on ice (4 × 45 sec sonication, 45 sec rest in between), using a SONICS VibraCell sonicator 150 sonicator, with a microtip at 60% amplitude. DNAse I (0.02 mg/mL) and $MgCl_2$ (5 mM) were added to the lysate to digest chromosomal DNA. The solution was left on ice for 20 minutes and then centrifuged (9,000 rpm, 20 minutes, 4ºC). The supernatant was retained for the assessment of protein levels or for the purification of soluble proteins. Enzymes were purified at 4ºC using an AKTA Fast Protein Liquid Chromatography (FPLC) System, Amersham Pharmacia Biotech (Liquid Chromatography Controller UPC-900 equipped with a high precision P-920 pump). The FPLC system was controlled from a PC running UNICORN v. 4.00. All buffers were freshly prepared with ddH_2O and filtered through 0.2 μm Millipore filter before use. The cell extracts were prepared in Protino Ni-TED lysis-equilibration-wash (LWE) buffer (0.5 mM NaCl, 20 mM Tris·HCl, pH 7.5). The crude cell lysate was filtered through a Millipore 0.45 μm filter before application on a TED (Tris-carboxymethyl ethylene diamine) column. The cleared lysates were then loaded onto a column pre-equilibrated with 10× column volume LEW buffer (1 CV = ~ 10 mL), followed by another 10 CV column wash. Protein was finally eluted with ~ 2 CV of elution buffer (0.5 mM NaCl, 0.5 mM imidazole, 20 mM Tris·HCl, pH 7.5). This procedure yielded His_6-tagged proteins of > 90% purity by SDS-PAGE. The protein was concentrated by ultrafiltration (10 kDa MW cut-off, Amicon ultra-15, Millipore) and subjected to thrombin cleavage. High grade thrombin (Novagen) was used to remove the His_6-tag from the *N*-terminus of a fusion protein according to the standard protocol: an accurate amount of thrombin (1 U per mg of protein) plus thrombin cleavage buffer were added to the protein solution as per the manufacturer's instructions. The thrombin-protein mixture was incubated at 4ºC overnight and then resolved by size exclusion chromatography. A Superdex S75, 300 mL (Pharmacia) column was fixed to the Akta FPLC and equilibrated with 1 CV of water and 2 CV of buffer (100 mM Tris·HCl, 100 mM NaCl, pH 7.5) at a flow rate of 2 mL/min. The proteins were concentrated to around 2 mL using an Amicon ultra-15, Millipore centrifugal filter membrane, and loaded onto the column using a 2mL/ 10mL loop at 0.5 mL/min. After loading of proteins, the flow rate was increased to 2 mL/min with the UV baseline corrected to zero. The protein fractions were collected by an automatic fraction collector when the UV peak level rose above 25 mAU, in typical fractions of 5 mL. The eluted fractions were analysed for purity by SDS-PAGE. Those fractions containg pure protein were pooled and buffer exchanged into 50 mM Tris·HCl pH 7.5 using a PD-10 desalting column (Amersham Biosciences) and stored at -80ºC.

1.1.9 Synthesis

All chemicals, reagents, and solvents were from Sigma-Aldrich (Dorset, UK) and used without further purification. HPLC grade solvents were used for reactions, chromatography, and work-ups. Aqueous solutions were made using de-ionized water. Thin layer chromatography (TLC) was carried out using Merck (Darmstadt, Germany) silica gel 60 F254 TLC plates. TLC visualisation was carried out under UV light and stained with one of three stains: ninhydrin, potassium permanganate, or anisaldehyde. Chromatographic purifications were carried out using a Biotage® (Uppsala, Sweden) Isolera One or Biotage® SP4 flash purification system, using Biotage® pre-packed SNAP columns. Reactions were monitored using an Agilent (Cheshire, UK) 1200 series, 6120 quadrupole LC-MS system using a Merck Chromolith® Performance RP-18 HPLC column. Deuterated solvents were obtained from Sigma-Aldrich, and ^{1}H NMR spectra were obtained on a Bruker AVANCE AVIII HD 400 nanobay (400 MHz), Bruker AV500 (500M Hz) with a ^{13}C cryoprobe, and/or Bruker AVIII 700 (700 MHz) with an inverse TCI cryoprobe. All signals are described in δ ppm with multiplets being denoted as singlet, doublet, triplet, quartet, and multiplet using the abbreviations s, d, t, q, and m, respectively. Chemical shifts in presented NMR spectra were referenced using residual solvent peaks with coupling constants, *J*, reported in hertz (Hz) to an accuracy of 0.5 Hz. For high-resolution mass spectrometry (HR-MS), a Bruker MicroTOF instrument with an ESI source and Time of Flight (TOF) analyser was used. MS data are represented as a ratio of mass to charge (m/z) in Daltons. A Bruker Tensor 27 instrument was used to obtain Fourier transform infrared spectra (FT-IR). Spectroscopic grade solvents and a Perkin Elmer 241 Polarimeter were used to obtain optical rotations.

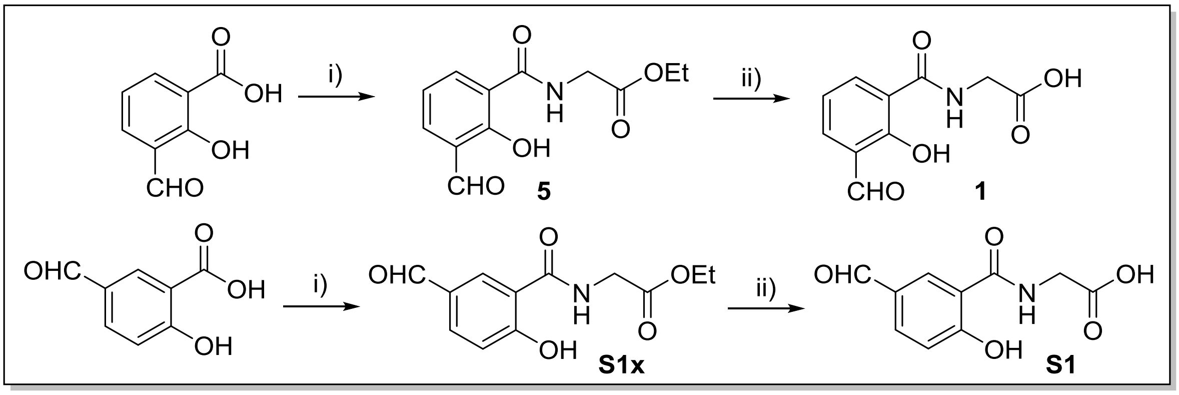

Scheme 1 *Reagents and conditions*: i) $EtOOCCH_2NH_3Cl$, EDCI, HOBT, Et_3N, DMF, MW, 6 min, 90ºC, 26-60%; ii) NaOH, H_2O, 2 hrs, rt, 90%.

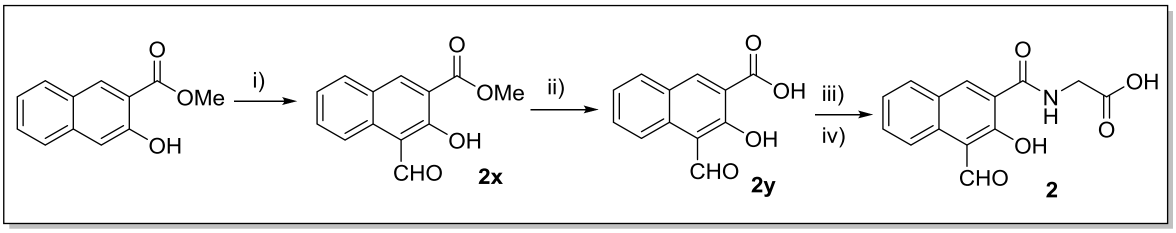

Scheme 2 *Reagents and conditions*: i) Cl_2CHOMe, $TiCl_4$, CH_2Cl_2, 2 hrs, reflux, 83%; ii) NaOH, H_2O, 2hrs, reflux, 94%; iii) $EtOOCCH_2NH_3Cl$, EDCI, HOBT, Et_3N, DMF, MW, 6 min, 90ºC; iv) NaOH, H_2O, 2 hrs, rt, 84%.

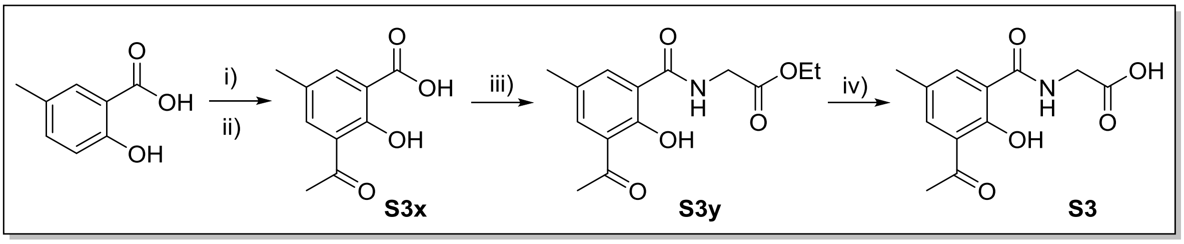

Scheme 3 *Reagents and conditions*: i) Ac_2O, catalytic conc.H_2SO_4, 2 hrs, reflux; ii) $AlCl_3$, 3 hrs, 180ºC; iii) $EtOOCCH_2NH_3Cl$, EDCI, HOBT, Et_3N, DMF, MW, 6 min, 90ºC, 44%; iv) NaOH, H_2O, 2 hrs, rt, 87%.

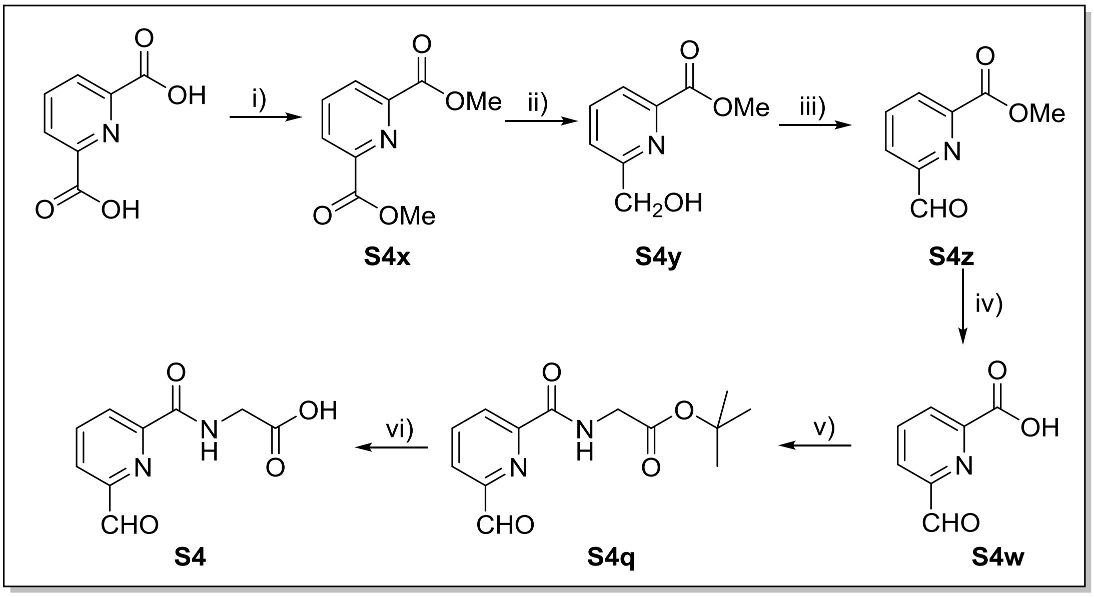

Scheme 4 *Reagents and conditions*: i) MeOH, catalytic conc. H_2SO_4, 48 hrs, reflux, 82%; ii) $NaBH_4$, $CaCl_2$, THF:MeOH 1:2, 3 hrs, 0ºC, 61%; iii) MnO_2, $CHCl_3$, 4 hrs, reflux, 36%; iv) NaOH, H_2O:MeOH 1:1, 2 hrs, rt, 74%; v) $tBuOOCCH_2NH_3Cl$, EDCI, HOBT, Et_3N, CH_2Cl_2, 16 hrs, rt, 37%; vi) TFA, CH_2Cl_2, 7 hrs, rt, 90%.

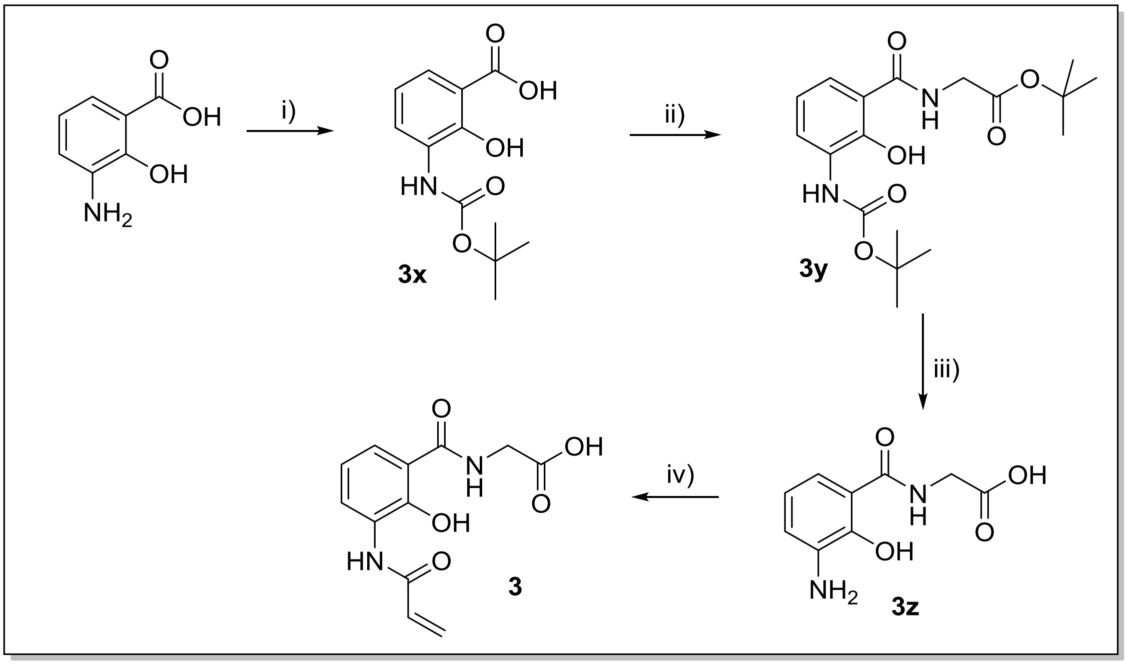

Scheme 5 *Reagents and conditions*: i) Boc_2O, Et_3N, CH_3CN, 24 hrs, rt; ii) $tBuOOCCH_2NH_3Cl$, EDCI, HOBT, Et_3N, DMF, MW, 6 min, 90ºC, 69%; iii) TFA, CH_2Cl_2, 7 hrs, rt, 99%; iv) acryloyl chloride, NaOH, H_2O, 16 hrs, 0ºC, 72%.

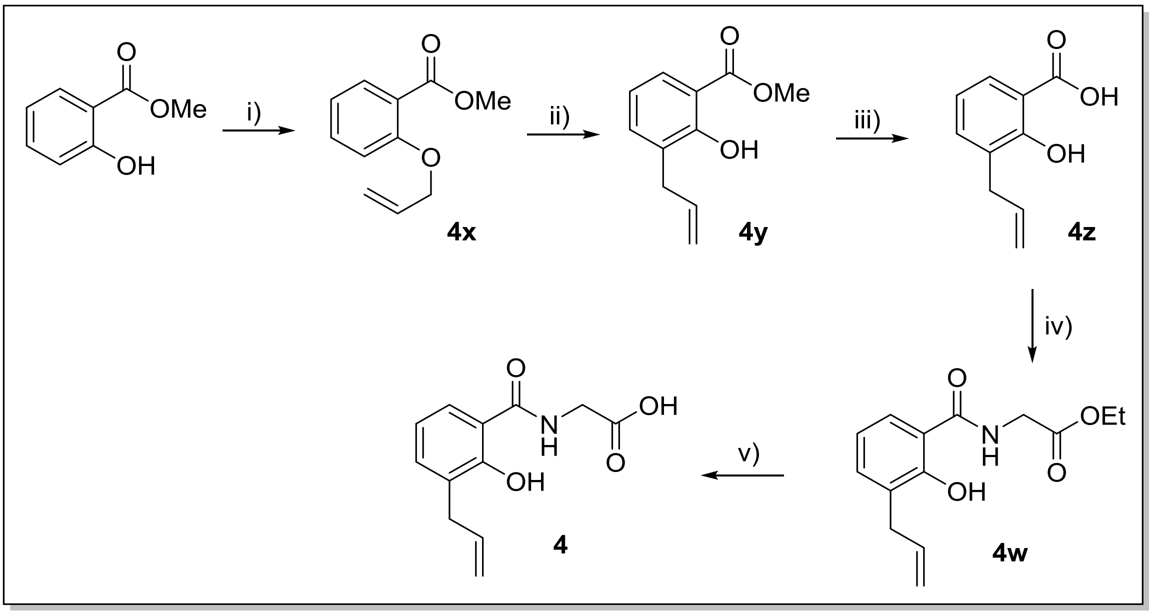

Scheme 6 *Reagents and conditions*: i) allyl bromide, K_2CO_3, acetone, 48 hrs, reflux, 54%; ii) 15 hrs, 200ºC, 77%; iii) NaOH, H_2O, 2 hrs, 50ºC, 92%; iv) $EtOOCCH_2NH_3Cl$, EDCI, HOBT, Et_3N, DMF, MW, 6 min, 90ºC, 86%; v) NaOH, H_2O, 2 hrs, rt, 95%.

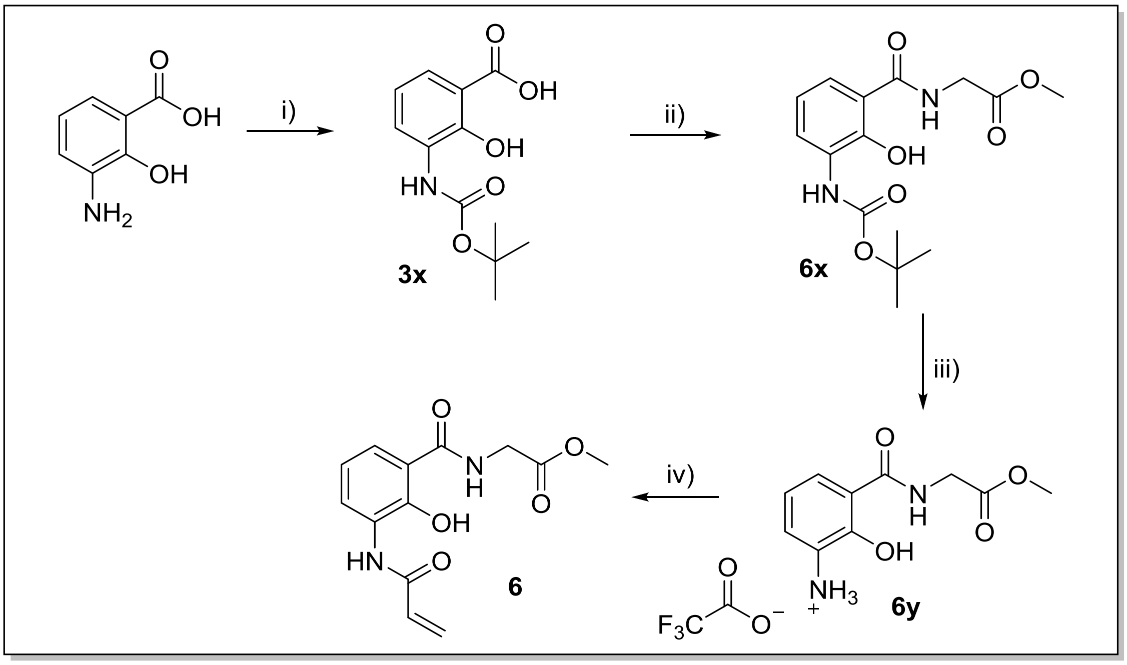

Scheme 7 *Reagents and conditions*: i) Boc_2O, Et_3N, CH_3CN, 24 hrs, rt; ii) $MeOOCCH_2NH_3Cl$, EDCI, HOBT, Et_3N, DMF, MW, 6 min, 90ºC, 61%; iii) TFA, CH_2Cl_2, 1.5 hrs, rt, 98%; iv) acryloyl chloride, NaOH, H_2O, 2 hrs, 0ºC, 94%.

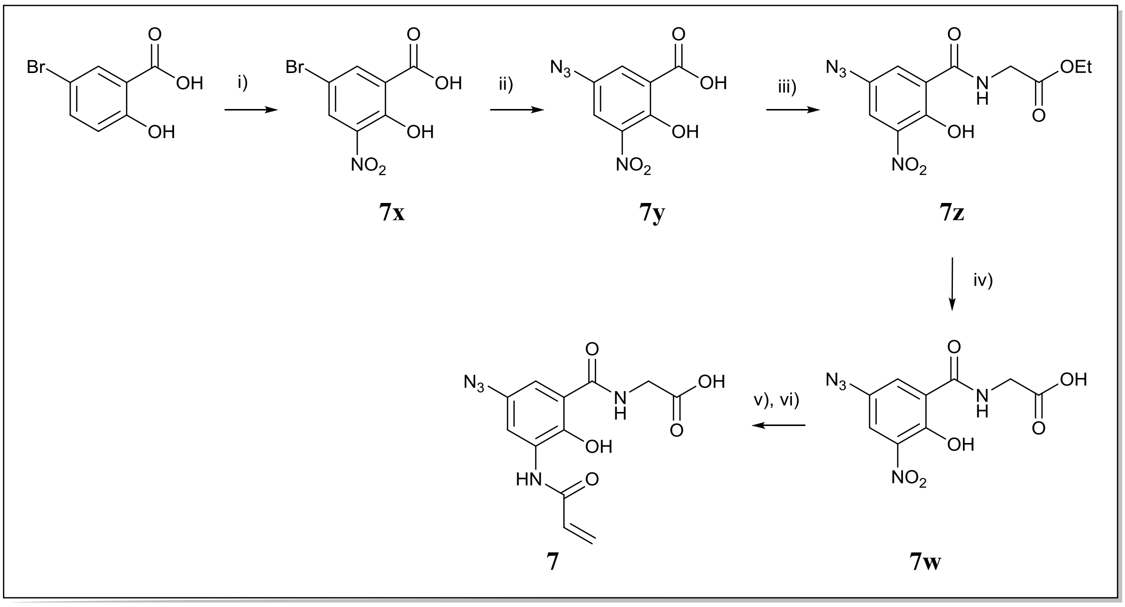

Scheme 8 *Reagents and conditions*: i) conc. H_2SO_4, HNO_3 (neat), 0°C, 2 hrs, 82%; ii) NaN_3 CuI, (R,R)-(-)-*N,N'*-Dimethyl-1,2-cyclohexanediamine, *L*-ascorbate, NaOH, EtOH/H_2O, 100°C, 40 mins, MW, 65%; iii) Glycine ethylester HCl, HATU, DBU, DCM, r.t., 3 hrs, 43%; iv) NaOH, THF, r.t., 2 hrs, 98%; v) $Na_2S_2O_4$(aq), THF, r.t., 15 mins; vi) Acrolyl chloride, NaOH, H_2O, 0°C, 1 hr, 25% (over two steps).

Ethyl 2-(3-formyl-2-hydroxybenzamido)acetate (5)

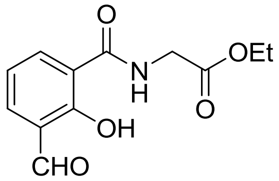

A mixture of 3-formylsalicylic acid (166 mg, 1 mmol), glycine ethyl ester hydrochloride (140 mg, 1 mmol), EDCI (230 mg, 1.2 mmol), HOBT (162 mg, 1.2 mmol) and Et_3N (121 mg, 1.2 mmol) in anhydrous DMF (3 mL) was irradiated under microwave (90ºC) for 6 minutes. Then the reaction mixture was diluted with 1N HCl (10 mL) and extracted with EtOAc (3 × 10 mL). Combined organic extracts were washed with H_2O (2 × 10 mL) and dried over $MgSO_4$. The crude product was purified by column chromatography (PE : EA 9:1 to 5:5) to afford 65 mg (26%) of **5** as a white solid.

m.p. 78-80ºC; FT-IR (KBr): 3360, 1733, 1683, 1645, 1598, 1554, 1224 cm^{-1}; ^1H NMR (200 MHz, $CDCl_3$): δ 1.30 (3H, t, J = 7.0 Hz, CH_3), 4.23-4.27 (4H, m, NCH_2 + OCH_2), 7.14 (1H, t, J = 8.0 Hz, ArCH), 7.75 (1H, dd, J = 2.0 Hz, 8.0 Hz, ArCH), 8.39 (2H, dd, J = 2.0 Hz, 8.0 Hz, ArCH + NH), 10.00 (1H, s, CHO), 12.64 (1H, s, OH) ppm; ^{13}C NMR (50 MHz, $CDCl_3$): δ 14.6 (CH_3), 42.4 (NCH_2), 62.0 (OCH_2), 120.4 (ArC), 120.6 (ArCH), 121.9 (ArC), 137.7 (ArCH), 139.4 (ArCH), 160.8 (C), 164.9 (C), 170.4 (C), 196.5 (CHO) ppm; HRMS (ESI$^+$) calcd. for $C_{12}H_{13}NNaO_5$ $(M+Na)^+$, 274.0686; found, 274.0688.

2-(3-Formyl-2-hydroxybenzamido)acetic acid (1)

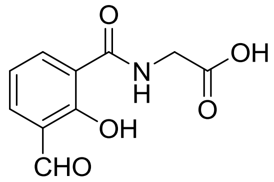

A solution of **5** (140 mg, 0.56 mmol) in 1N NaOH (1.7 mL, 1.7 mmol) was stirred under Ar at room temperature for 2 hours. The solution was then acidified with 6N HCl to pH 2 and extracted with EtOAc (3 × 30 mL), dried over $MgSO_4$ and evaporated to afford 122 mg (90%) of **1** as a white solid.

m.p. 198-202ºC; FT-IR (KBr): 3405, 1744, 1656, 1558, 1211 cm^{-1}; ^{1}H NMR (500 MHz, DMSO-d_6): δ 4.03 (2H, d, *J* = 5.5 Hz, CH_2), 7.10 (1H, t, *J* = 8.0 Hz, ArCH), 7.90 (1H, dd, *J* = 2.0 Hz, 8.0 Hz, ArCH), 8.21 (1H, dd, *J* = 2.0 Hz, 8.0 Hz, ArCH), 9.49 (1H, t, *J* = 5.5 Hz, NH), 10.37 (1H, s, CHO), 12.84 (1H, bs, OH), 13.71 (1H, bs, OH) ppm; ^{13}C NMR (125 MHz, DMSO-d_6): δ 41.0 (CH_2), 115.8 (ArC), 118.8 (ArCH), 123.9 (ArC), 132.8 (ArCH), 134.0 (ArCH), 163.3 (C), 169.3 (C), 170.5 (C), 189.4 (CHO) ppm; HRMS (ESI^-) calcd. for $C_{10}H_8NO_5$ $(M-H)^-$, 222.0408; found, 222.0412.

Ethyl 2-(5-formyl-2-hydroxybenzamido)acetate (S1x)

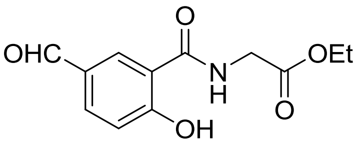

A mixture of 5-formylsalicylic acid (166 mg, 1 mmol), glycine ethyl ester hydrochloride (140 mg, 1 mmol), EDCI (230 mg, 1.2 mmol), HOBT (162 mg, 1.2 mmol) and Et_3N (121 mg, 1.2 mmol) in anhydrous DMF (3 mL) was irradiated under microwave (90ºC) for 6 minutes. Then the reaction mixture was diluted with 1N HCl (10 mL) and extracted with EtOAc (3 × 10 mL). Combined organic extracts were washed with H_2O (2 × 10 mL) and dried over $MgSO_4$. The crude product was purified by column chromatography (PE : EA 9:1 to 5:5) to afford 151 mg (60%) of **S1x** as a white solid.

m.p. 123-124ºC; FT-IR (KBr): 3337, 1734, 1676, 1645, 1583, 1550, 1216 cm^{-1}; ^{1}H NMR (500 MHz, $CDCl_3$): δ 1.35 (3H, t, *J* = 7.0 Hz, CH_3), 4.25 (2H, d, *J* = 4.5 Hz, NCH_2), 4.31 (2H, q, *J* = 7.0 Hz, OCH_2), 7.11 (1H, d, *J* = 8.5 Hz, ArCH), 7.15 (1H, t, *J* = 4.5 Hz, NH), 7.93 (1H, dd, *J* = 2.0 Hz, 8.5 Hz, ArCH), 8.07 (1H, d, *J* = 2.0 Hz, ArCH), 9.88 (1H, s, CHO), 12.85 (1H, s, OH) ppm; ^{13}C NMR (125 MHz, $CDCl_3$): δ 14.1 (CH_3), 41.5 (NCH_2), 62.1 (OCH_2), 114.0 (ArC), 119.4 (ArCH), 127.9 (ArC), 128.0 (ArCH), 136.0 (ArCH), 166.8 (C), 169.3 (C), 169.3 (C), 189.9 (CHO) ppm; HRMS (ESI^+) calcd. for $C_{12}H_{13}NNaO_5$ $(M+Na)^+$, 274.0686; found, 274.0683.

2-(5-Formyl-2-hydroxybenzamido)acetic acid (S1)

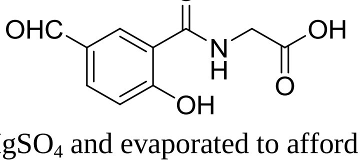

A solution of **S1x** (140 mg, 0.56 mmol) in 1N NaOH (1.7 mL, 1.7 mmol) was stirred under Ar at room temperature for 2 hours. Then it was acidified with 6N HCl to pH 2 and extracted with EtOAc (3 × 30 mL), dried over $MgSO_4$ and evaporated to afford 122 mg (90%) of **S1** as a white solid.

m.p. 207-210ºC; FT-IR (KBr): 3381, 1728, 1685, 1671, 1645, 1490 cm^{-1}; ^{1}H NMR (500 MHz, DMSO-d_6): δ 4.03 (2H, d, *J* = 5.5 Hz, NCH_2), 7.14 (1H, d, *J* = 8.5 Hz, ArCH), 7.97 (1H, dd, *J* = 2.0 Hz, 8.0 Hz, ArCH), 8.51 (1H, d, *J* = 2.0 Hz, ArCH), 9.34 (1H, t, *J* = 5.5 Hz, NH), 9.87 (1H, s, CHO), 12.84 (1H, bs, OH), 13.08 (1H, bs, OH) ppm; ^{13}C NMR (125 Hz, DMSO-d_6): δ 41.2 (CH_2), 116.2 (ArC), 118.2 (ArCH), 128.1 (ArCH), 131.8 (ArCH), 134.4 (ArCH), 164.2 (C), 167.4 (C), 170.8 (C), 190.9 (CHO) ppm; HRMS (ESI^-) calcd. for $C_{10}H_8NO_5$ $(M-H)^-$, 222.0408; found, 222.0415.

Methyl 4-formyl-3-hydroxy-2-naphthoate (2x)

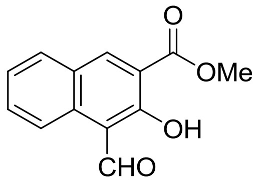

2x was prepared following the reported procedure.[7]

To a stirred solution of methyl 3-hydroxy-2-naphthoate (2.02 g, 10.0 mmol) in anhydrous CH_2Cl_2 (30 mL) were added 1M $TiCl_4$ (16.7 mL, 16.7 mmol) and Cl_2CHOMe (3.48 g, 30.3 mmol). The reaction mixture was then refluxed for 2 hours, cooled to 0ºC and carefully quenched with H_2O. After washing with H_2O, the organic phase was dried over Na_2SO_4, filtered and evaporated. The remaining residue was purified by column chromatography (PE : EA 9:1 to 5:5) to afford 1.91 g (83%) of **2x** as a yellow solid.

m.p. 138-139ºC (lit. 141-142ºC)[16]; ^{1}H NMR (500 MHz, $CDCl_3$): δ 4.05 (3H, s, OCH_3), 7.41-7.45 (1H, m, ArCH), 7.68-7.71 (1H, m, ArCH), 7.80 (1H, d, *J* = 8.0 Hz, ArCH), 8.64 (1H, s, ArCH), 9.09 (1H, d, *J* = 8.0 Hz, ArCH), 10.90 (1H, s, CHO), 11.97 (1H, bs, OH) ppm; ^{13}C NMR (125 MHz, $CDCl_3$): δ 53.0 (OCH_3), 123.6 (ArCH), 125.1 (ArCH), 126.4 (ArC), 130.1 (ArCH), 132.5 (ArCH), 134.6 (ArC), 140.5 (ArCH), 163.7 (C), 169.0 (C), 191.6 (CHO) ppm; HRMS (ESI^+) calcd. for $C_{13}H_{10}NaO_5$ $(M+Na)^+$, 253.0471; found, 253.0475.

4-Formyl-3-hydroxy-2-naphthoic acid (2y)

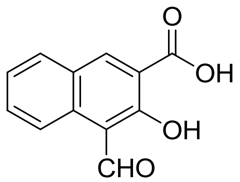

A stirred solution of **2x** (607 mg, 2.64 mmol) in 1N NaOH (8.0 mL, 8.0 mmol) was under reflux for 2 hours under Ar. Then it was cooled to room temperature, acidified with 6N HCl to pH 3 and the formed precipitation was collected and washed with H_2O to afford 534 mg (94%) of **2y** as a yellow solid.

m.p. 224-227ºC; ^{1}H NMR (500 MHz, DMSO-d_6): δ 7.49 (1H, t, *J* = 8.0 Hz, ArCH), 7.75 (1H, t, *J* = 8.0 Hz, ArCH), 8.09 (1H, d, *J* = 8.0 Hz, ArCH), 8.86 (1H, s, ArCH), 9.10 (1H, d, *J* = 8.0 Hz, ArCH), 10.8 (1H, s, CHO) ppm; ^{13}C NMR (125 MHz, DMSO-d_6): δ 113.9 (ArC), 115.8 (ArC), 123.3 (ArCH), 124.8 (ArCH), 126.1 (ArC), 130.5 (ArCH), 132.3 (ArCH), 133.6 (ArC), 140.5 (ArCH), 164.9 (ArC), 171.2 (COO), 190.8 (CHO) ppm; HRMS (ESI^-) calcd. for $C_{12}H_7O_4$ $(M-H)^-$, 215.0350; found, 215.0353.

2-(4-Formyl-3-hydroxy-2-naphthamido)acetic acid (2)

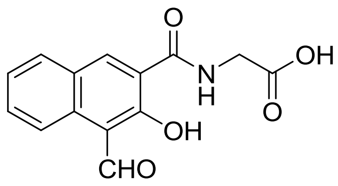

A mixture of **2y** (432 mg, 2.0 mmol), glycine ethyl ester hydrochloride (307 mg, 2.2 mmol), EDCI (460 mg, 2.4 mmol), HOBT (324 mg, 2.4 mmol) and Et_3N (243 mg, 2.4 mmol) in anhydrous DMF (5 mL) was irradiated under microwave (90ºC) for 6 minutes. Then the reaction mixture was diluted with 1N HCl (20 mL) and extracted with EtOAc (3 × 20 mL). Combined organic extracts were washed with H_2O (2 × 20 mL) and dried over $MgSO_4$. The crude product was purified by column chromatography (PE : EA 5:5) to afford 421 mg (70%) of ethylester of **6** as a yellow solid. To a solution of this ester (85 mg, 0.28 mmol) in EtOH (1 mL) was then added a solution of NaOH (34 mg, 0.85 mmol) in H_2O. The resulting mixture was stirred for 2 hours under Ar. Then EtOH was removed under reduced pressure and the remaining residue acidified with 1N HCl to pH 3. The formed precipitation was collected and washed with H_2O to afford 65 mg (84%) of **2** as an orange solid.

m.p. 198-200ºC; FT-IR (KBr): 3379 (bs), 1733, 1650, 1539, 1212 cm^{-1}; ^{1}H NMR (500 MHz, DMSO-d_6): δ 4.10 (2H, d, *J* = 5.5 Hz, CH_2), 7.53 (1H, t, *J* = 8.0 Hz, ArCH), 7.77 (1H, t, *J* = 8.0 Hz, ArCH), 7.98 (1H, d, *J* = 8.0 Hz, ArCH), 8.90 (1H, s, ArCH), 9.03 (1H, d, *J* = 8.0 Hz, ArCH), 9.65 (1H, t, *J* = 5.5 Hz, NH), 10.83 (1H, s, CHO), 12.89 (1H, bs, OH), 14.09 (1H, bs, COOH) ppm; ^{13}C NMR (125 MHz, DMSO-d_6): δ 41.3 (CH_2), 113.7 (ArC), 117.2 (ArC), 122.7 (ArCH), 125.1 (ArCH), 126.0 (ArC), 130.1 (ArCH), 131.8 (ArCH), 133.0 (ArC), 137.9 (ArCH), 164.2 (ArC), 168.3 (CO), 170.5 (CO), 192.0 (CHO) ppm; HRMS (ESI^-) calcd. for $C_{14}H_{10}NO_5$ (M-H), 272.0564; found, 272.0567.

3-Acetyl-2-hydroxy-5-methylbenzoic acid (S3x)

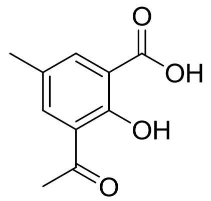

S3x was prepared following the reported procedure.[8]

To a suspension of 5-methylsalicylic acid (3.04 g, 20 mmol) in acetic anhydride (15 mL) was added a catalytic amount of conc. H_2SO_4 (30 μL). The resulting solution was refluxed for 2 hours, and then cooled to room temperature. Water was added carefully, and the precipitate collected by filtration, and washed with H_2O to provide 3.24 g (84%) of 2-acetoxy-5-methylsalicylic acid as a white solid. 2-Acetoxy-5-methylsalicylic acid (700 mg, 3.61 mmol) and $AlCl_3$ (1.44 g, 10.8 mmol) were heated under Ar at 180ºC for 3 hours. Then, the mixture was cooled to room temperature and poured into ice/conc. HCl (30 g, 8 mL). EtOAc (15 mL) was then added and the mixture extracted with CH_2Cl_2 (3 × 60 mL). Combined extracts were dried over $MgSO_4$, filtered and evaporated to afford 610 mg (87%) of **S3x** as a creamy yellow solid.

^{1}H NMR (400 MHz, $CDCl_3$): δ 2.37 (3H, s, $ArCH_3$), 2.71 (3H, s, $COCH_3$), 7.81 (1H, d, *J* = 2.0 Hz, ArCH), 8.15 (1H, d, *J* = 2.0 Hz, ArCH), 9.91 (1H, bs, XH), 14.06 (1H, bs, XH) ppm; ^{13}C NMR (100 MHz, $CDCl_3$): δ

20.2 (ArCH$_3$), 27.8 (CO**C**H$_3$), 116.1 (ArC), 121.1 (ArC), 129.1 (ArC), 136.8 (ArCH), 139.7 (ArCH), 158.4 (ArC-OH), 167.2 (CO), 204.2 (**C**OCH$_3$) ppm.

Ethyl 2-(3-acetyl-2-hydroxy-5-methylbenzamido)acetate (S3y)

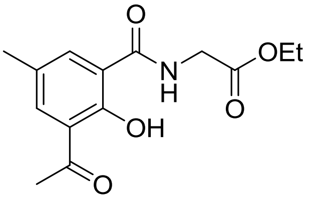

A mixture of **S3x** (388 mg, 2.0 mmol), glycine ethyl ester hydrochloride (307 mg, 2.2 mmol), EDCI (460 mg, 2.4 mmol), HOBT (324 mg, 2.4 mmol) and Et$_3$N (243 mg, 2.4 mmol) in anhydrous DMF (5 mL) was irradiated under microwave (90ºC) for 6 minutes. Then the reaction mixture was diluted with 1N HCl (20 mL) and extracted with EtOAc (3 × 20 mL). Combined organic extracts were washed with H$_2$O (2 × 20 mL) and dried over MgSO$_4$. The crude product was purified by column chromatography (PE : EA 5:5) to afford 245 mg (44%) of **S3y** as a yellow solid.

m.p. 101-103ºC; ^{1}H NMR (500 MHz, CDCl$_3$): δ 1.32 (3H, t, *J* = 7.5 Hz, CH$_2$C**H**$_3$), 2.37 (3H, s, ArCH$_3$), 2.69 (3H, s, COCH$_3$), 4.24-4.28 (4H, m, 2 × CH$_2$), 7.72 (1H, d, *J* = 2.0 Hz, ArCH), 8.29 (1H, d, *J* = 2.0 Hz, ArCH), 8.71 (1H, bs, NH), 13.87 (1H, s, OH) ppm; ^{13}C NMR (125 MHz, CDCl$_3$): δ 14.2 (CH$_2$**C**H$_3$), 20.4 (ArCH$_3$), 27.0 (CO**C**H$_3$), 42.0 (NCH$_2$), 61.5 (OCH$_2$), 119.8 (ArC), 120.6 (ArC), 128.4 (ArC), 134.7 (ArCH), 140.1 (ArCH), 159.0 (ArC-OH), 164.6 (CO), 170.0 (CO), 205.3 (**C**OCH$_3$) ppm; HRMS (ESI$^+$) calcd. for $C_{17}H_{17}NNaO_5$ $(M+Na)^+$, 302.0999; found, 302.1008.

2-(3-Acetyl-2-hydroxy-5-methylbenzamido)acetic acid (S3)

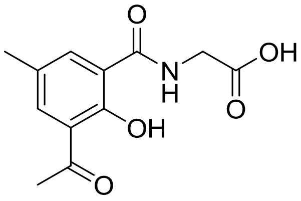

A solution of **S3y** (112 mg, 0.4 mmol) in 1N NaOH (1.2 mL, 1.2 mmol) was stirred at room temperature for 2 hours under Ar. Then it was acidified with 1N HCl to pH 3 and the formed precipitate was collected and washed with H$_2$O to afford 88 mg (87%) of **S3** as a white solid.

m.p. 242-245ºC; ^{1}H NMR (500 MHz, DMSO-d$_6$): δ 2.32 (ArCH$_3$), 2.67 (COCH$_3$), 4.02 (2H, d, *J* = 5.5 Hz, NCH$_2$), 7.85 (1H, d, *J* = 2.0 Hz, ArCH), 7.99 (1H, d, *J* = 2.0 Hz, ArCH), 8.99 (1H, t, *J* = 5.5 Hz, NH), 12.76 (1H, bs, OH), 13.71 (1H, s, OH) ppm; ^{13}C NMR (125 MHz, DMSO-d$_6$): δ 19.9 (ArCH$_3$), 29.2 (CO**C**H$_3$), 41.3 (NCH$_2$), 118.1 (ArC), 123.0 (ArC), 127.5 (ArC), 135.1 (ArCH), 135.5 (ArCH), 158.3 (ArC), 166.7 (CO), 170.8 (CO), 202.8 (**C**OCH$_3$) ppm; HRMS (ESI$^-$) calcd. for $C_{12}H_{12}NO_5$ $(M-H)^-$, 250.0721; found, 250.0724.

Dimethyl pyridine-2,6-dicarboxylate (S4x)

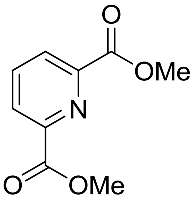

To a solution of pyridine-2,6-dicarboxylic acid (4.00 g, 24.0 mmol) in MeOH (40 mL) was added dropwise concentrated H$_2$SO$_4$ (4 mL, 75.4 mmol). The mixture was then

heated to reflux for 46 hrs. The reaction mixture was cooled to room temperature and the methanol concentrated in vacuo. The aqueous layer was then carefully neutralized using a saturated solution of $NaHCO_3$. The aqueous phase was extracted with $CHCl_3$ (3 × 50 mL) and combined organic extracts were washed with H_2O (2 × 50 mL), brine (50 mL), dried over $MgSO_4$ and concentrated *in vacuo* to afford **S4x** as a white solid (3.83 g, 82%).

R_f = 0.50 (silica gel, EtOAc:PE 1:1); m.p. 117-119°C; ^{1}H NMR (200 MHz, $CDCl_3$): δ 4.03 (6H, s, 2 × $COOCH_3$), 8.03 (1H, t, *J* =8.0 Hz, ArCH), 8.32 (2H, d, *J* = 8.0 Hz, 2 × ArCH) ppm.

Methyl 6-(hydroxymethyl)pyridine-2-carboxylate (S4y)

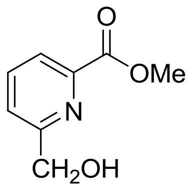

S4y was prepared following the reported procedure.[9]

To a slurry of compound **S4x** (585 mg, 3.00 mmol) and $CaCl_2$ (1.33 g, 12 mmol) in THF:MeOH (10 mL:20 mL) at 0°C under nitrogen was carefully added $NaBH_4$ (284 mg, 7.50 mmol) in portions. The resulting mixture was stirred at 0°C for 20 min and then poured into ice-water (50 mL), extracted with $CHCl_3$ (3 × 30 mL), dried over $MgSO_4$ and concentrated in vacuo to afford 306 mg (61%) of **S4y** as a white crystalline solid.

R_f = 0.30 (silica gel, EtOAc:CH_2Cl_2 1:1); m.p. 83-85°C; FT-IR (film): 3439 (br), 1730, 1594, 1443, 1317, 1139 cm^{-1}; ^{1}H NMR (400 MHz, $CDCl_3$): δ 3.87 (1H, t, *J* = 5.5 Hz, OH), 3.99 (3H, s, $COOCH_3$), 4.86 (2H, d, *J* = 5.5 Hz, CH_2), 7.55 (1H, d, *J* = 8.0 Hz, ArCH), 7.84 (1H, dd, *J* = 8.0 Hz, 8.0 Hz, ArCH), 8.01 (1H, d, *J* = 8.0 Hz, ArCH) ppm; ^{13}C NMR (400 MHz, $CDCl_3$): δ 52.9 (OCH_3), 64.6 (CH_2OH), 123.8 (ArCH), 124.1 (ArCH), 137.7 (ArCH), 146.9 (ArC), 160.4 (ArC), 165.6 (COO) ppm; HRMS (FI$^+$) calcd for $C_8H_9NO_3$ (M), 167.0582; found, 167.0586.

Methyl 6-(formyl)pyridine-2-carboxylate (S4z)

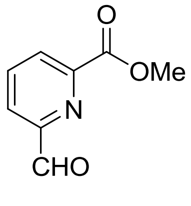

To a solution of compound **S4y** (589 mg, 3.52 mmol) in $CHCl_3$ (25 mL) was added manganese dioxide (6.00 g, 69 mmol) and the mixture was heated to reflux for 5 hrs. The reaction mixture was then cooled down and filtered through a pad of silica gel, washed with EtOAc and evaporated affording **S4z** as a white solid (212 mg, 36%).

R_f = 0.25 (silica gel, CH_2Cl_2); m.p. 94-97°C; FT-IR (film): ^{1}H NMR (400 MHz, $CDCl_3$): δ 4.05 (3H, s, $COOCH_3$), 8.05 (1H, dd, *J* = 8.0 Hz, 8.0 Hz, ArCH), 8.14 (1H, dd, *J* = 1.0 Hz, 8.0 Hz, ArCH), 8.34 (1H, dd, *J* = 1.0 Hz, 8.0 Hz, ArCH), 10.17 (1H, s, CHO) ppm; ^{13}C NMR (400 MHz, $CDCl_3$): δ 53.3 (OCH_3), 124.4 (ArCH), 129.1 (ArCH), 138.4 (ArCH), 164.8 (COO), 192.6 (CHO) ppm; HRMS (FI$^+$) calcd for $C_8H_7NO_3$ (M), 165.0426; found, 165.0426.

6-Formylpyridine-2-carboxylic acid (S4w)

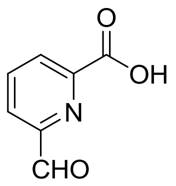

To a solution of compound **S4z** (101 mg, 0.61 mmol) in THF:H_2O 1:1 (8 mL) was added 1M NaOH (5 mL) and the mixture was stirred for 30 min at room temperature under Ar. Then THF was evaporated, the aqeuous layer acidified with 1M HCl (6 mL) and extracted with EtOAc (6 × 15 mL), dried over $MgSO_4$ and concentrated in vacuo to afford **S4w** as a white solid (68 mg, 74 %).

R_f = 0.10 (silica gel, EtOAc:MeOH:AcOH 80:20:2); m.p. 163-167°C; FT-IR (KBr): 3062 (br), 1714, 1702, 1329, 1291, 1223 cm^{-1}; ^1H NMR (400 MHz, DMSO-d_6): δ 8.10 (1H, dd, *J* = 1.0 Hz, 8.0 Hz, ArCH), 8.21 (1H, dd, *J* = 8.0 Hz, 8.0 Hz, ArCH), 8.30 (1H, dd, *J* = 1.0 Hz, 8.0 Hz, ArCH), 10.02 (1H, s, CHO), 13.61 (1H, bs, COOH); ^{13}C NMR (400 MHz, DMSO-d_6): δ 125.1 (ArCH), 129.7 (ArCH), 140.1 (ArCH), 149.9 (ArC), 153.1 (ArC), 166.4 (COOH), 194.0 (CHO) ppm.

***tert*-Butyl 2-(6-formylpicolinamido)acetate (S4q)**

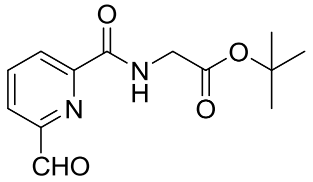

To a solution of acid **S4w** (227 mg, 1.5 mmol) in $CHCl_3$ (15 mL) were added *tert*-butyl glycine hydrochloride (277 mg, 1.65 mmol), HOBT (223 mg, 1.65 mmol), EDCI (316 mg, 1.65 mmol) and Et_3N (379 mg, 3.75 mmol). The resulting solution was then stirred at room temperature for 16 hours. After washing with sat. $NaHCO_3$ (2 × 15 mL) and water (15 mL), the organic phase was dried over $MgSO_4$ and evaporated. Purification by flash chromatography (petroleum ether 40-60°C/EtOAc 8:2 to 5:5) to yield 145 mg (37%) of **S4q** as a colourless oil.

^1H NMR (400 MHz, $CDCl_3$): δ 1.50 (9H, s, $C(CH_3)_3$), 4.19 (2H, d, *J* = 5.5 Hz, CH_2), 8.02-8.09 (2H, m, 2 × ArCH), 8.40 (1H, dd, *J* = 1.5 Hz, 7,5 Hz, ArCH), 8.47 (1H, t, *J* = 5.5 Hz, NH), 10.08 (1H, s, CHO) ppm; ^{13}C NMR (100 MHz, $CDCl_3$): δ 28.0 (C(**C**$H_3)_3$), 42.0 (CH_2), 82.5 (**C**$(CH_3)_3$), 123.8 (ArCH), 126.4 (ArCH), 138.7 (ArCH), 149.9 (ArC), 151.3 (ArC), 163.3 (CO), 168.7 (CO), 192.4 (CHO) ppm; HRMS (ESI^-) calcd. for $C_{13}H_{15}N_2O_4$ $(M-H)^-$, 263.1037; found, 263.1027.

2-(6-Formylpicolinamido)acetic acid (S4)

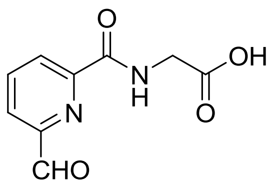

To a stirred solution of **S4q** (110 mg, 0.42 mmol) in CH_2Cl_2 (5 mL) at 0ºC was added CF_3COOH (0.8 mL). The reaction mixture was stirred at room temperature for 7 hours, then solvent was evaporated and the residue then coevaporated with Et_2O (10 × 5 mL) to yield 78 mg (90%) of **S4** as a white solid.

m.p. 128-133ºC; ^{1}H NMR (500 MHz, DMSO-d$_6$): δ 4.05 (2H, d, *J* = 6.0 Hz, CH_2), 8.15 (1H, dd, *J* = 1.5 Hz, 8.0 Hz, ArCH), 8.27 (1H, t, *J* = 8.0 Hz, ArCH), 8.33 (1H, dd, *J* = 1.5 Hz, 8.0 Hz, ArCH), 9.12 (1H, t, *J* = 6.0 Hz, NH), 10.06 (1H, s, CHO), 12.74 (1H, bs, COOH) ppm; ^{13}C NMR (125 MHz, DMSO-d$_6$): δ 41.1 (CH_2), 124.3 (ArCH), 126.2 (ArCH), 139.7 (ArCH), 150.0 (ArC), 151.2 (ArC), 163.4 (CO), 171.0 (CO), 192.7 (CHO) ppm; HRMS (ESI$^-$) calcd. for $C_9H_7N_2O_5$ $(M-H)^-$, 207.0411; found, 207.0412.

3-(*tert*-Butoxycarbonylamino)-2-hydroxybenzoic acid (3x)

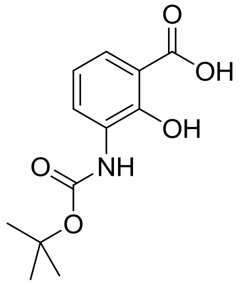

To an ice-cooled solution of 3-aminosalicylic acid (765 mg, 5.0 mmol) in dioxane/water (14 mL/7 mL) were added Et_3N (1.05 mL, 7.5 mmol) and di-*tert*-butyl dicarbonate (1.64 g, 7.5 mmol). The resulting mixture was then stirred for 24 hours at room temperature. The solvent was then removed under reduced pressure and HCl (3N) was slowly added to the crude residue. The resulting precipitate was then collected by filtration to afford 1.24 g (98 %) of **3x** as a white solid.

m.p. 107-111ºC; ^{1}H NMR (400 MHz, DMSO-d$_6$): δ 1.45 ($C(CH_3)_3$), 6.87 (1H, t, *J* = 8.0 Hz, ArCH), 7.50 (1H, d, *J* = 8.0 Hz, ArCH), 7.86 (1H, d, *J* = 8.0 Hz, ArCH), 8.07 (1H, s, XH) ppm; ^{13}C NMR (100 MHz, DMSO-d$_6$): δ 28.9 (C(**C**$H_3)_3$), 80.3 (**C**$(CH_3)_3$), 113.4 (ArC), 119.3 (ArCH), 125.2 (ArCH), 127.7 (ArCH), 127.9 (ArC), 153.3 (ArC or CO), 153.7 (ArC or CO), 173.2 (CO) ppm; HRMS (ESI$^+$) calcd for $C_{12}H_{15}NNaO_5$ $(M+Na)^+$ 276.0842; found 276.0847.

***tert*-Butyl 2-(3-(*tert*-butoxycarbonylamino)-2-hydroxybenzamido)acetate (3y)**

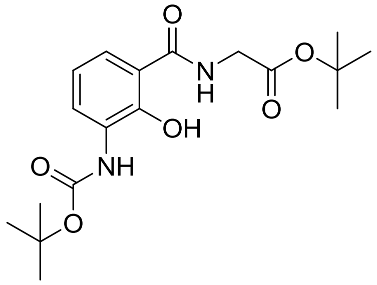

A mixture of **3x** (1.01 g, 4.0 mmol), glycine *tert*-butyl ester hydrochloride (737 mg, 4.4 mmol), EDCI (920 mg, 4.8 mmol), HOBT (648 mg, 4.8 mmol) and Et_3N (485 mg, 4.8 mmol) in anhydrous DMF (12 mL) was irradiated under microwave (90ºC) for 6 minutes. Then the reaction mixture was diluted with 1N HCl (20 mL) and extracted with EtOAc (3 × 20 mL). Combined organic extracts were washed with H_2O (2 × 20 mL) and dried over $MgSO_4$. The crude product was purified by column chromatography (PE : EA 9:1 to 7:3) to afford 1.02 g (69%) of **3y** as a white solid.

^{1}H NMR (400 MHz, $CDCl_3$): δ 1.52 (9H, s, $C(CH_3)_3$), 1.53 (9H, s, $C(CH_3)_3$), 4.11 (2H, d, *J* = 5.0 Hz, NCH_2), 6.84 (1H, t, *J* = 8.0 Hz, ArCH), 6.93 (1H, t, *J* = 5.0 Hz, N**H**CH_2), 7.09 (1H, d, *J* = 8.0 Hz, ArCH), 7.21 (1H,

bs, ArNH), 8.21 (1H, d, *J* = 8.0 Hz, ArCH), 12.73 (1H, s, OH) ppm; ^{13}C NMR (100 MHz, $CDCl_3$): δ 28.0 ($C(\mathbf{C}H_3)_3$), 28.3 ($C(\mathbf{C}H_3)_3$), 42.1 (NCH_2), 80.5 ($\mathbf{C}(CH_3)_3$), 83.1 ($\mathbf{C}(CH_3)_3$), 112.7 (ArC), 118.2 (ArCH), 118.7 (ArCH), 121.9 (ArCH), 128.7 (ArC), 150.2 (C), 152.8 (C), 168.6 (CO), 169.9 (CO) ppm; HRMS (ESI$^+$) calcd for $C_{18}H_{26}N_2NaO_6$ $(M+Na)^+$ 389.1683; found 389.1680.

2-(3-Amino-2-hydroxybenzamido)acetic acid (3z)

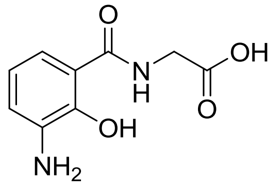

To a stirred solution of **3y** (831 mg, 2.27 mmol) in CH_2Cl_2 (15 mL) at 0ºC was added CF_3COOH (2.5 mL). The reaction mixture was stirred at room temperature for 8 hours, then solvent was evaporated and the residue then coevaporated with Et_2O (10 × 10 mL) to yield 730 mg (99%) of **3z** as a white solid.

m.p. 136-139ºC; FT-IR (KBr): 3357, 1738, 1663, 1202 cm^{-1}; ^{1}H NMR (500 MHz, DMSO-d_6): δ 3.98 (2H, d, *J* = 6.0 Hz, NCH_2), 6.83 (1H, t, *J* = 8.0 Hz, ArCH), 7.14 (1H, d, *J* = 8.0 Hz, ArCH), 7.48 (1H, d, *J* = 8.0 Hz, ArCH), 7.32 (1H, t, *J* = 6.0 Hz, NH) ppm; ^{13}C NMR (125 MHz, DMSO-d_6): δ 41.0 (NCH_2), 113.7 (ArC), 114.9 (ArC), 118.5 (ArCH), 119.7 (ArCH), 122.2 (ArCH), 151.3 (ArC), 170.4 (CO), 170.7 (CO) ppm; HRMS (ESI$^-$) calcd. for $C_9H_9N_2O_4$ $(M-H)^-$, 209.0568; found, 209.0571.

2-(3-Acrylamido-2-hydroxybenzamido)acetic acid (3)

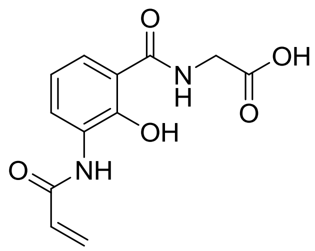

To a stirred solution of **3z** (84 mg, 0.4 mmol) and NaOH (16 mg, 0.4 mmol) in H_2O (6 mL) at 0ºC was added acryloyl chloride (177 mg, 1.96 mmol). The reaction mixture was then stirred at 0ºC for 12 hours, and the formed precipitate was collected and washed with water to afford 76 mg (72%) of **3** as a violet solid.

m.p. 199-201ºC; FT-IR (KBr): 3402, 1721, 1640, 1550, 1228 cm^{-1}; ^{1}H NMR (500 MHz, DMSO-d_6): δ 4.00 (2H, d, *J* = 5.5 Hz, NCH_2), 5.75 (1H, dd, *J* = 2.0 Hz, 10.0 Hz, =CH), 6.26 (1H, dd, *J* = 2.0 Hz, 17.0 Hz, =CH), 6.76 (1H, d, *J* = 10.0 Hz, 17.0 Hz, CO-CH=), 6.92 (1H, t, *J* = 8.0 Hz, ArCH), 7.67 (1H, d, *J* = 8.0 Hz, ArCH), 8.22 (1H, d, *J* = 8.0 Hz, ArCH), 9.38 (1H, t, *J* = 5.5 Hz, N**H**CH_2), 9.57 (1H, bs, NH), 12.79 (1H, bs OH), 13.43 (1H, s, OH) ppm; ^{13}C NMR (125 MHz, DMSO-d_6): δ 41.0 (NCH_2), 113.8 (ArC), 118.0 (ArCH), 122.2 (ArCH), 126.0 (ArCH), 127.0 (=CH_2), 127.3 (ArC), 131.9 (CO-**C**H=), 152.1 (ArC), 163.6 (CO), 170.2 (CO), 170.7 (CO) ppm; HRMS (ESI$^+$) calcd. for $C_{12}H_{12}N_2NaO_5$ $(M+Na)^+$, 287.0638; found, 287.0642.

Methyl 2-(allyloxy)benzoate (4x)

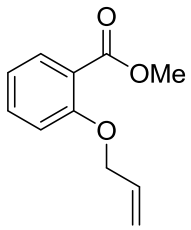

4x was prepared following the reported procedure.[10]

A mixture of methyl salicylate (7.61 g, 50 mmol), allyl bromide (9.08 g, 75 mmol) and potassium carbonate (8.29 g, 60 mmol) in acetone (30 mL) was refluxed for 48 hours. Then it was cooled to room temperature and the solid residue was filtered. The filtrate was evaporated and resulting residue purified by column chromatography (PE to PE: EA 9:1) to afford 5.21 g (54%) of **4x** as a colourless oil.

^{1}H NMR (500 MHz, $CDCl_3$): δ 3.91 (3H, s, OCH_3), 4.63-4.65 (2H, m, OCH_2), 5.31 (1H, dd, *J* = 1.5 Hz, 10.5 Hz, =CH), 5.52 (1H, dd, *J* = 1.5 Hz, 17.5 Hz, =CH), 6.04 (1H, m, –CH=), 6.96-7.01 (2H, m, 2 × ArCH), 7.43-7.46 (1H, m, ArCH), 7.81 (1H, dd, *J* = 2.0 Hz, 8.0 Hz, ArCH) ppm; ^{13}C NMR (125 MHz, $CDCl_3$): δ 52.0 (OCH_3), 69.4 (OCH_2), 113.6 (ArCH), 117.4 (=CH_2), 120.4 (ArCH), 120.5 (ArC), 131.7 (ArCH), 132.7 (–CH=), 133.4 (ArCH), 158.1 (ArC), 166.8 (COO) ppm; HRMS (ESI$^+$) calcd for $C_{11}H_{12}NaO_3$ $(M+Na)^+$ 215.0679; found 215.0687.

Methyl 3-allyl-2-hydroxybenzoate (4y)

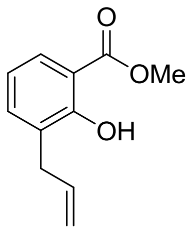

4x was heated at 200ºC under N_2 for 15 hours. Purification by column chromatography (PE : EA 9:1) afforded 2.02 g (77%) of **4y** as a white solid.

^{1}H NMR (500 MHz, $CDCl_3$): δ 3.45 (2H, d, *J* = 6.5 Hz, $ArCH_2$), 3.95 (3H, s, OCH_3), 5.07-5.11 (2H, m, =CH_2), 5.98-6.06 (1H, m, –CH=), 6.84 (1H, t, *J* = 7.5 Hz, ArCH), 7.34 (1H, dd, *J* = 1.5 Hz, 8.0 Hz, ArCH), 7.73 (1H, dd, *J* = 1.5 Hz, 8.0 Hz, ArCH), 11.06 (1H, s, OH) ppm; ^{13}C NMR (125 MHz, $CDCl_3$): δ 33.7 ($ArCH_2$), 52.3 (OCH_3), 111.9 (ArC), 115.9 (=CH_2), 118.7 (ArCH), 127.9 (ArCH), 128.5 (ArC), 135.8 (ArCH), 136.2 (–CH=), 159.6 (ArC), 171.0 (COO) ppm; HRMS (ESI$^-$) calcd. for $C_{11}H_{11}O_3$ $(M-H)^-$, 191.0714; found, 191.0718.

3-Allyl-2-hydroxybenzoic acid (4z)

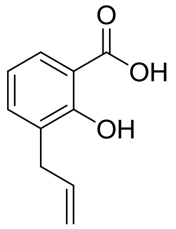

A stirred solution of **4y** (768 mg, 4.0 mmol) in 1N NaOH (12.0 mL, 12.0 mmol) was heated at 50ºC for 2 hours under Ar. Then it was cooled to room temperature, acidified with 6N HCl to pH 3 and the formed precipitate was collected and washed with H_2O to afford 652 mg (92%) of **4z** as a white solid.

m.p. 81-83ºC; ^{1}H NMR (500 MHz, DMSO-d_6): δ 3.37 (2H, d, *J* = 6.5 Hz, $ArCH_2$), 5.04-5.08 (2H, m, =CH_2), 5.94-6.02 (1H, m, –CH=), 6.88 (1H, t, *J* = 7.5 Hz, ArCH), 7.38 (1H, dd, *J* = 2.0 Hz, 7.5 Hz, ArCH), 7.69 (1H, dd, *J* = 2.0 Hz, 7.5 Hz, ArCH), 11.66 (1H, bs, OH), 13.99 (1H, bs, COOH) ppm; ^{13}C NMR (125 MHz, DMSO-d_6): δ 33.1 ($ArCH_2$), 112.2 (ArC), 116.0 (=CH_2), 118.7 (ArCH), 127.7 (ArC), 128.3

(ArCH), 135.6 (ArCH), 136.3 (–CH=), 159.2 (ArC), 172.5 (COO) ppm; HRMS (ESI^-) calcd. for $C_{10}H_9O_3$ (M-H)$^-$, 177.0557; found, 177.0561.

Ethyl 2-(3-allyl-2-hydroxybenzamido)acetate (4w)

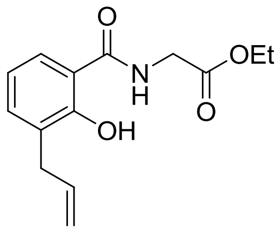

A mixture of **4z** (712 mg, 4.0 mmol), glycine ethyl ester hydrochloride (614 mg, 4.4 mmol), EDCI (649 mg, 4.8 mmol), HOBT (920 mg, 4.8 mmol) and Et_3N (485 mg, 4.8 mmol) in anhydrous DMF (10 mL) was irradiated under microwave (90ºC) for 6 minutes. Then the reaction mixture was diluted with 1N HCl (10 mL) and extracted with EtOAc (3 × 10 mL). Combined organic extracts were washed with H_2O (2 × 10 mL) and dried over $MgSO_4$. The crude product was purified by column chromatography (PE : EA 8:2 to 6:4) to afford 908 mg (86%) of **4w** as a white solid.

m.p. 71-74ºC; ^{1}H NMR (400 MHz, $CDCl_3$): δ 1.33 (3H, t, *J* = 7.5 Hz, CH_3), 3.43 (2H, d, *J* = 6.5 Hz, $ArCH_2$), 4.21 (2H, d, *J* = 5.0 Hz, NCH_2), 4.29 (2H, q, *J* = 7.5 Hz, OCH_2), 5.06-5.12 (2H, m, $=CH_2$), 5.97-6.07 (1H, m, –CH=), 6.81 (1H, t, *J* = 7.5 Hz, ArCH), 6.93 (1H, t, *J* = 5.0 Hz, NH), 7.29 (1H, d, *J* = 7.5 Hz, ArCH), 7.34 (1H, d, *J* = 7.5 Hz, ArCH), 12.32 (1H, s, OH) ppm; ^{13}C NMR (100 MHz, $CDCl_3$): δ 14.1 (CH_3), 33.7 ($ArCH_2$), 41.5 (NCH_2), 61.9 (OCH_2), 113.4 (ArC), 115.9 ($=CH_2$), 118.3 (ArCH), 123.7 (ArCH), 129.5 (ArC), 134.5 (ArCH), 136.2 (–CH=), 159.5 (ArC), 169.8 (CO), 170.3 (CO) ppm; HRMS (ESI^+) calcd for $C_{14}H_{17}NNaO_4$ $(M+Na)^+$, 286.1050; found 286.1046.

2-(3-Allyl-2-hydroxybenzamido)acetic acid (4)

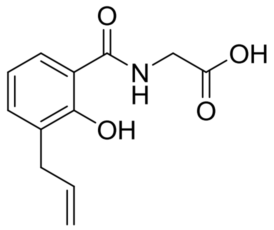

To a stirred solution of **4w** (460 mg, 1.75 mmol) in MeOH (3 mL) was added a solution of 1N NaOH (5.25 mL, 5.25 mmol) in H_2O. The resulting mixture was then stirred for 1 hour under N_2. Then EtOH was removed under reduced pressure and the remaining residue acidified with 1N HCl to pH 3. The formed precipitate was collected and washed with H_2O to afford 390 mg (95%) of **4** as a white solid.

m.p. 129-131ºC; ^{1}H NMR (500 MHz, DMSO-d_6): δ 3.35 (2H, d, *J* = 6.5 Hz, $ArCH_2$), 3.97 (2H, d, *J* = 5.5 Hz, NCH_2), 5.03-5.08 (2H, m, $=CH_2$), 5.93-6.01 (1H, m, –CH=), 6.87 (1H, t, *J* = 7.5 Hz, ArCH), 7.32 (1H, dd, *J* = 1.5 Hz, 7.5 Hz, ArCH), 7.75 (1H, dd, *J* = 1.5 Hz, 7.5 Hz, ArCH), 9.29 (1H, t, *J* = 5.5 Hz, NH), 12.78 (1H, bs, OH), 13.04 (1H, bs, OH) ppm; ^{13}C NMR (125 MHz, DMSO-d_6): δ 33.2 ($ArCH_2$), 41.0 (NCH_2), 113.4 (ArC), 115.8 ($=CH_2$), 118.1 (ArCH), 125.1 (ArCH), 128.2 (ArC), 134.1 (ArCH), 136.4 (–CH=), 158.9 (ArC), 170.6 (CO), 170.8 (CO) ppm; HRMS (ESI^+) calcd for $C_{12}H_{13}NNaO_4$ $(M+Na)^+$ 258.0737; found 258.0728.

2-hydroxy-3-((*O*-methylglycine)- *N*-*tert*butyl-benzocarbamate (6x)

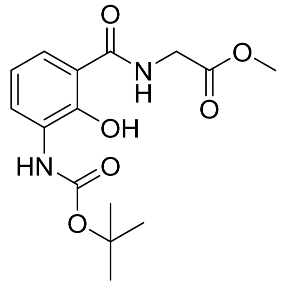

To a solution of 3-(terbutoxycarbonyl) amino salicylic acid (506 mg, 2.00 mmol, 1.0 equiv) in DMF (4 mL, anhydrous) were added HOBt (390.5 mg, 2.4 mmol 1.2 equiv), EDCI·HCl (554 mg, 2.4 mmol, 1.2 equiv), H-Gly-OMe HCl (333 mg, 2.2 mmol, 1.1 equiv) and triethylamine (405 μL, 2.4 mmol, 1.2 equiv). The mixture was charged with a stirring bar and the now dark brown suspension was irradiated in the microwave for 6 min at 90 °C. The dark brown viscous solution was washed 3x with H_2O in EtOAc. The organic phase was dried with Na_2SO_4 and after filtration the crude was obtained *in vacuo*. Crude product was purified by column chromatography [SiO_2, 50 gram] 30% to 40% EtOAc : n-Heptane over 500 mL. The combined fractions were furnished under reduced pressure after 3x toluene and 3x $CHCl_3$ azeotropic removal to afford **6x** as a white solid (395 mg, 61%). ESI-MS in MeCN, $[M+Na]^+$ calc: 347.1 found: 347.0. ESI HR-MS in MeCN, $[M+Na]^+$ calc: 347.1219, found: 347.1222. ^{1}H NMR (400 MHz, $CDCl_3$): δ 12.61 (s, 1 H, Ph-O*H*), 8.22 (d, *J*= 7.5 Hz, 1 H, 4-Ph-H), 7.21 (bs, 1 H, PhN*H*Boc), 7.09 (dd, *J*= 8 Hz 1.6 Hz, 1 H, 6-Ph-H) ,6.91 (bt, *J*= 5.0 Hz, 1 H, Ph-CON*H*CH_2), 6.89 (dt, *J*= 8 Hz 0.5 Hz, 1 H, 5-Ph-H), 4.23 (d, *J*= 5.0 Hz, 1 H, NHCH_2COO), 3.83 (s, 3 H, COOCH_3), 1.53 (s, 9 H, COC(CH_3)$_3$).

2-hydroxy-3-((2-methoxy-2-oxoethyl)carbamoyl)benzenaminium 2,2,2-trifluoroacetate (6y)

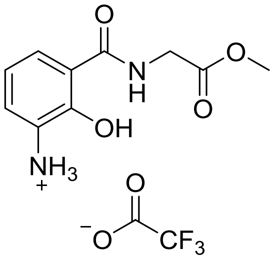

To a yellow to orange solution of **6x** (275 mg, 1.18 mmol, 1.0 equiv.) in CH_2Cl_2 (12 mL) was added TFA (3 mL, 39.20 mmol, 33 equiv.) dropwise while mechanically stirred. The mixture monitored by TLC and ESI-MS and after 1.5 h volatiles were removed *in vacuo*. Azeotropic removal with 3x toluene and 3x Et_2O yielded **6y** as a white solid of (258 mg, 98% yield). ESI-MS in MeOH, $[M+H]^+$ calc: 225.1 found: 225.0. ^{1}H NMR (400 MHz, DMSO-d_6): δ 9.43 (bt, *J*= 4.5 Hz, 1 H, CON*H*CH_2), 7.49 (d, *J*= 6.5 Hz, 1 H, 6-Ph-*H*), 7.15 (d, *J*= 6 Hz, 1 H, 4-Ph-*H*), 6.84 (t, *J*= 6.5 Hz, 1 H, 5-Ph-*H*), 4.08 (d, *J*= 5.0 Hz, 2 H, NHCH_2COO), 3.69 (s, 3 H, $COOCH_3$).

2-hydroxy-3-((*O*-methylglycine)- *N*-benzoacrylamide (6)

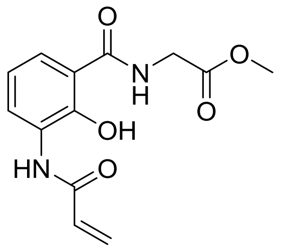

A suspension of **6y** (50 mg, 0.148 mmol, 1.0 equiv.) in CH_2Cl_2 (4 mL, anhydrous) was cooled down to 0 °C while mechanically stirred for 10 min followed by dropwise addition of Et_3N (22.8 μL, 0.163 mmol, 1.1 equiv). After 10 min, acyloyl chloride (18 μL, 0.222 mmol, 1.5 equiv) was added dropwise maintaining set conditions. The mixture was allowed to gradually warm up to room temperature, stirred for 2 hours and the crude was extracted from a 1 N HCl solution (15 mL), 3x 15 mL CH_2Cl_2. The organic phases combined were dried over Na_2SO_4 and evaporated to afford **6** (37 mg, 94% yield) as a white solid. ESI-MS in MeOH, $[M+H]^+$ calc: 301.1 found: 301.0. ^{1}H NMR (400 MHz, $CDCl_3$, Acetone-d_6 (5:3): δ 12.80 (s, 1 H, Ph-O*H*), 8.22 (d, *J*= 8.0 Hz, 1 H, 4-Ph-H), 8.00 (bs, 1 H, PhN*H*), 7.20 (dd,

J= 8.5, 1.5 Hz, 1 H, 6-Ph-H), 6.97 (bs, 1 H, Ph-CON*H*CH_2), 6.91 (t, J= 8 Hz, 1 H, 5-Ph-H), 6.44 (dd, J= 17, 1 Hz, 1 H, NH-CH=CHH, 6.32 (dd, J= 17, 10 Hz, 1 H, N*H*-CH=C*H*H), 5.79 (dd, J= 10, 1.5 Hz, 1 H, NH-CH=CH*H*), 4.24 (d, J= 5.0 Hz, 1 H, NHC$\underline{H_2}$COO), 3.87 (s, 3 H, COOC$\underline{H_3}$). ^{13}C NMR (125 MHz, DMSO-d_6): δ 170.3, 169.8, 163.6, 152.0, 131.8, 127.3, 127.0, 126.2, 122.2, 118.0, 113.7, 51.2, 41.0.

5-Bromo-2-hydroxy-3-nitrobenzoic acid (7x)

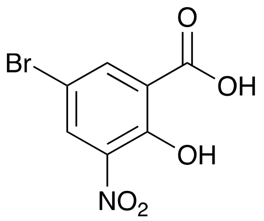

To a suspension of 5-bromosalicylic acid (23 g, 105 mmol) in concentrated sulfuric acid (70 ml) at 0°C, a mixture of nitric acid (6.6 ml) and conc. sulfuric acid (15.4 ml) was added dropwise whilst maintaining a reaction temperature of < 10°C. The reaction was stirred on ice for 1 hour before the ice bath was removed and the reaction stirred at room temperature for 3 hours at which point the reaction was observed to be complete by LCMS. The resulting mixture was decanted into a conical flask containing crushed ice, which gave a bright yellow precipitate which was collected by büchner filtration, washed with cold water and dried to leave a yellow solid. After recrystallised from EtOAc, a crystalline bright yellow solid **7x** was obtained (22.4 g, 86.1 mmol, 82%).

m.p. 168-170°C. ^{1}H NMR (400 MHz, CD_3OD) δ 8.26 (1H, d, J = 2.5 Hz), 8.28 (1H, d, J = 2.5 Hz) ppm. ^{13}C NMR (101 MHz, CD_3OD) δ 110.1, 118.7, 126.6, 134.3, 138.8, 155.8, 171.4 ppm. FT-IR ν_{max} (neat) 2581, 1673, 1522, 1428, 1341, 1231, 1172, 1153, 954, 906, 895, 795, 709, 692 cm^{-1}. HRMS (ESI-TOF) calcd for $C_7H_4BrNO_5$ $[M+H]^+$: 259.9200 and 261.9180, found: 259.9198 and 261.9177.

5-Azido-2-hydroxy-3-nitrobenzoic acid (7y)

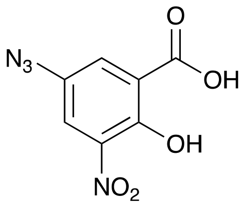

To a solution of 5-bromo-2-hydroxy-3-nitrobenzoic acid (**7x**) (1.35 g, 5.15 mmol) in a 5:1 mixture of ethanol and distilled water (10.3 ml) was added sodium azide (670 mg, 10.3 mmol), sodium *L*-ascorbate (51.4 mg, 0.26 mmol), copper(I) iodide (98 mg, 0.52 mg) and (*R*,*R*)-(−)-*N*,*N'*-dimethyl-1,2-cyclohexanediamine. The reaction vessel was placed under an atmosphere of N_2 before being heated to 100°C with stirring using a microwave reactor, until complete consumption of the starting material was observed by LCMS (~ 45 mins). The reaction was cooled to room temperature before being quenched with 1.0M $HCl_{(aq)}$ (~ 10 ml). The resulting suspension was passed through a short pad of silica gel and the filtrate was extracted with EtOAc (3 x 20 ml). The organic layers were collected, dried over anhydrous $MgSO_4$, filtered and concentrated before being purified by flash column chromatography (99:1, EtOAc/AcOH). The appropriate fractions were collected and concentrated to give **7y** as a dark yellow solid. (798 mg, 3.58 mmol, 65%).

m.p. 154-156°C. ^{1}H NMR (400 MHz, DMSO-d_6) δ 7.73 (1H, d, J = 3.0 Hz), 7.89 (1H, d, J = 3.0 Hz) ppm. ^{13}C NMR (101 MHz, DMSO-d_6) δ 120.9, 125.3, 129.1, 137.2, 138.6, 152.7, 169.8 ppm. FT-IR ν_{max} (neat) 3362, 3016, 2970, 2497, 1738, 1532, 1448, 1365, 1229, 1216, 1168, 889, 796 cm^{-1}. HRMS (ESI-TOF) calcd for $C_7H_3N_4O_5$ $[M-H]^-$: 223.0109, found: 223.0108.

Ethyl (5-azido-2-hydroxy-3-nitrobenzoyl)glycinate (7z)

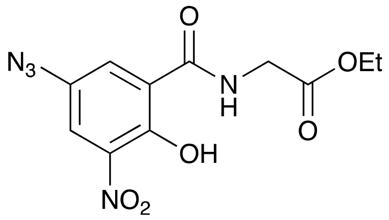

5-Azido-2-hydroxy-3-nitrobenzoic acid (**7y**) (525 mg, 2.34 mmol), HATU (1.78 g, 4.69 mmol), glycine ethyl ester hydrochloride salt (359 mg, 2.57 mmol) and DBU (1.8 ml, 11.7 mmol) were dissolved in CH_2Cl_2 (23ml). The mixture was stirred at rom temperature untill the reaction was observed to be complete by TLC (17:1, CH_2Cl_2/MeOH). Following addition 1M $HCl_{(aq)}$ (30 ml) the organic materials were extracted with CH_2Cl_2 (3 x 30 ml), after which all organic layers were combined, dried over anhydrous $MgSO_4$, filtered and concentrated. The crude material was then purified by flash column chromatography (0 to 60% EtOAc in cyclohexane 10 CVs). The appropriate fractions were collected and concentrated to give **7z** as a dark orange oil (310 mg, 1.0 mmol, 43% yield).

^{1}H NMR (400 MHz, CD_3OD) δ 1.29 (3H, t, *J* = 7.0 Hz), 4.16 (2H, s), 4.22 (2H, q, *J* = 7.0 Hz), 7.81 (1H, d, *J* = 3.0 Hz), 7.90 (1H, d, *J* = 3.0 Hz) ppm. ^{13}C NMR (101 MHz, CD_3OD) δ 14.5, 40.1, 62.5, 120.4, 120.7, 124.5, 126.2, 132.9, 152.3, 167.5, 170.9 ppm. FT-IR ν_{max} (film) 3410, 2940, 2110, 1742, 1650, 1583, 1535, 1470, 1377, 1325, 1210, 838 cm^{-1}. HRMS (ESI-TOF) calcd for $C_{11}H_{12}N_5O_6$ $[M+H]^+$: 310.0782, found: 310.0783.

(5-Azido-2-hydroxy-3-nitrobenzoyl)glycine (7w)

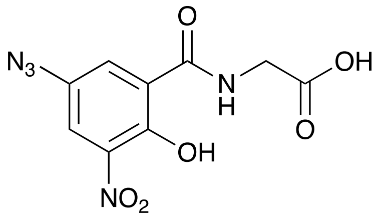

To a solution of (5-azido-2-hydroxy-3-nitrobenzoyl)glycinate (**7z**) (883 mg, 2.86 mmol) in THF (5.72 ml), 1.0M $NaOH_{(aq)}$ was added and after 30 minutes a red precipitate had formed which was isolated by büchner filtration, washed several times with CH_2Cl_2 and dried under vacuum flow to yield the product **7w** as a red soild (801 mg, 2.85 mmol, 99%).

m.p. 184-187°C. ^{1}H NMR (400 MHz, DMSO-d_6) δ 3.59 (2H, d, *J* = 4.5 Hz), 7.45 (1H, d, *J* = 3.5 Hz), 7.71 (1H, d, *J* = 3.5 Hz) ppm. ^{13}C NMR (101 MHz, DMSO-d_6) δ 44.8, 118.8, 118.9, 126.6, 127.2, 136.2, 164.4, 172.2 ppm. FT-IR ν_{max} (film) 3415, 2126, 1651, 1586, 1537, 1509, 1406, 1334, 1285, 1248, 838, 794 cm^{-1}. HRMS (ESI-TOF) calcd for $C_9H_6N_5O_6$ $[M-H]^-$: 280.0324, found: 280.0321.

(3-Acrylamido-5-azido-2-hydroxybenzoyl)glycine (7)

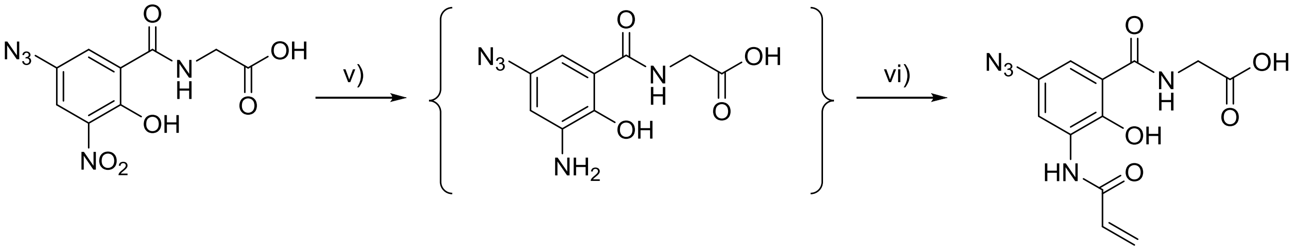

7w **7wa** **7**

Part v): To a solution of (5-azido-2-hydroxy-3-nitrobenzoyl)glycine (**7w**) (200 mg, 0.71 mmol) in THF (5.46 ml), a solution of $Na_2S_2O_4$ (1.24 g, 7.1 mmol) in distilled water (10.6 ml) was added dropwise. After 15 minutes the reaction was observed to be complete by LCMS and the reaction was decanted into a saturated brine solution. The mixture was extracted with EtOAc (5 x 30 ml) and the organic fractions collected, dried over anhydrous Na_2SO_4, filtered and concentrated to give amine (**7wa**) as a clear oil (100mg, 0.40 mmol, 56% yield), which was used without further purification for the next step.

Part vi): (3-amino-5-azido-2-hydroxybenzoyl)glycine (**7wa**) (100 mg, 0.40 mmol) was suspended in water (2 ml) and cooled with an ice bath. To this suspension was added 1.0M $NaOH_{(aq)}$ (400 μL) and acrolyl chloride (159 μL, 1.96 mmol). The mixture was then stirred for 1 hour after which a beige precipitate was observed to form which was isolated by Büchner filtration, dried over 48 hrs in a vacuum to give the product **7** as a beige solid (55 mg, 0.18 mmol, 25% yield over two steps).

m.p. (decomp.) 289-290°C. ^{1}H NMR (400 MHz, CD_3OD) δ 4.10 (2H, s), 5.80 (1H, dd, *J* = 10.5, 1.5 Hz), 6.39 (1H, dd, *J* = 17.0, 1.5 Hz), 6.60 (1H, dd, *J* = 17.0, 10.5 Hz), 7.27 (1H, d, *J* = 2.5 Hz), 8.16 (1H, d, *J* = 2.5 Hz) ppm. ^{13}C NMR (101 MHz, CD_3OD) δ 41.8, 113.1, 115.9, 116.9, 128.6, 129.9, 132.0, 132.1, 150.3, 166.4, 171.2, 172.8 ppm. FT-IR ν_{max} (film) 2928, 2496, 2115, 1728, 1643, 1595, 1538, 1475, 1447, 1247, 970, 792 cm^{-1}. HRMS (ESI-TOF) calcd for $C_{12}H_{12}N_5O_5$ $[M+H]^+$: 306.0833, found: 306.0835.

3. References

1. J. Mecinović, R. Chowdhury, B. M. R. Liénard, E. Flashman, M. R. G. Buck, N. J. Oldham and C. J. Schofield, *ChemMedChem*, 2008, 3, 569-572.
2. E. Flashman, L. M. Hoffart, R. B. Hamed, J. M. Bollinger, C. Krebs and C. J. Schofield, *FEBS J.*, 2010, **277**, 4089-4099.
3. R. Chowdhury, J. I. Candela-Lena, M. C. Chan, D. J. Greenald, K. K. Yeoh, Y. M. Tian, M. A. McDonough, A. Tumber, N. R. Rose, A. Conejo-Garcia, M. Demetriades, S. Mathavan, A. Kawamura, M. K. Lee, F. van Eeden, C. W. Pugh, P. J. Ratcliffe and C. J. Schofield, *ACS Chem. Biol.*, 2013, **8**, 1488–1496.
4. P. J. Boersema, R. Raijmakers, S. Lemeer, S. Mohammed and A. J. R. Heck, *Nat. Protocols*, 2009, **4**, 484–494.
5. N. Mischerikow, A. F. M. Altelaar, J. D. Navarro, S. Mohammed and A. J. R. Heck, *Mol. Cell. Proteomics*, 2010, **9**, 2140-2148
6. R. Chowdhury, M. A. McDonough, J. Mecinović, C. Loenarz, E. Flashman, K. S. Hewitson, C. Domene and C. J. Schofield, *Structure*, 2009, **17**, 981-989.
7. M. Ashram, S. Miyzed and P. E. Georghiou, *J. Org. Chem.*, 2011, **66**, 1473-1479.
8. G. Aromí, P. Gamez, O. Roubeau, P. Carrero Berzal, H. Kooijman‡, A. L. Spek, W. L. Driessen and J. Reedijk, *Inorg. Chem.*, 2002, **41**, 3673-3683.
9. C. Schmuck and U. Machon, *Chem. Eur. J.*, 2005, **11**, 1109-1118.
10. A. I. Meyers, M. Reuman and R. A. Gabel, *J. Org. Chem.*, 1981, **46**, 783-788.
